# Metastasis-suppressor NME1 controls the invasive switch of breast cancer by regulating MT1-MMP surface clearance

**DOI:** 10.1038/s41388-021-01826-1

**Published:** 2021-05-19

**Authors:** Catalina Lodillinsky, Laetitia Fuhrmann, Marie Irondelle, Olena Pylypenko, Xiao-Yan Li, Hélène Bonsang-Kitzis, Fabien Reyal, Sophie Vacher, Claire Calmel, Olivier De Wever, Ivan Bièche, Marie-Lise Lacombe, Ana Maria Eiján, Anne Houdusse, Anne Vincent-Salomon, Stephen J. Weiss, Philippe Chavrier, Mathieu Boissan

**Affiliations:** 1 Research Area, Instituto de Oncología Ángel H. Roffo, Universidad de Buenos Aires, Buenos Aires, Argentina; 2 Consejo Nacional de Investigaciones Científicas y Técnicas (CONICET), Buenos Aires, Argentina; 3 Diagnostic and Theranostic Medicine Division, Institut Curie, PSL Research University, Paris, France; 4 Institut Curie, CNRS UMR144, PSL Research University, Paris, France; 5 Division of Medical Genetics, Department of Internal Medicine, Life Sciences Institute, University of Michigan, Ann Arbor, MI USA; 6 Department of Surgery, Institut Curie, Paris, France; 7 Translational Research Department, RT2Lab Team, INSERM U932, Immunity and Cancer, Institut Curie, Paris, France; 8 Pharmacogenomic Unit, Institut Curie, Paris, France; 9 INSERM UMR_S 938, Saint-Antoine Research Center, CRSA, University Sorbonne, Paris, France; 10 Laboratory of Experimental Cancer Research, Cancer Research Institute Ghent (CRIG), Department of Human Structure and Repair, Ghent University, Ghent, Belgium; 11 Laboratory of Biochemistry and Hormonology, Tenon Hospital, AP-HP, Paris, France; 12 Present Address: Gynecological and Breast Surgery and Cancerology Center, RAMSAY-Générale de Santé, Hôpital Privé des Peupliers, Paris, France

**Keywords:** Breast cancer, Endocytosis

## Abstract

Membrane Type 1 Matrix Metalloprotease (MT1-MMP) contributes to the invasive progression of breast cancers by degrading extracellular matrix tissues. Nucleoside diphosphate kinase, NME1/NM23-H1, has been identified as a metastasis suppressor; however, its contribution to local invasion in breast cancer is not known. Here, we report that NME1 is up-regulated in ductal carcinoma in situ (DCIS) as compared to normal breast epithelial tissues. NME1 levels drop in microinvasive and invasive components of breast tumor cells relative to synchronous DCIS foci. We find a strong anti-correlation between NME1 and plasma membrane MT1-MMP levels in the invasive components of breast tumors, particularly in aggressive histological grade III and triple-negative breast cancers. Knockout of NME1 accelerates the invasive transition of breast tumors in the intraductal xenograft model. At the mechanistic level, we find that MT1-MMP, NME1 and dynamin-2, a GTPase known to require GTP production by NME1 for its membrane fission activity in the endocytic pathway, interact in clathrin-coated vesicles at the plasma membrane. Loss of NME1 function increases MT1-MMP surface levels by inhibiting endocytic clearance. As a consequence, the ECM degradation and invasive potentials of breast cancer cells are enhanced. This study identifies the down-modulation of NME1 as a potent driver of the in situ-to invasive transition during breast cancer progression.

## Introduction

Ductal carcinoma in situ (DCIS) correspond to the proliferation of neoplastic breast epithelial cells contained within a layer of myoepithelial cells and an intact basement membrane [1]. If untreated, some DCIS (20–50%) will progress to invasive breast cancer (IBC) with characteristic tumor cell dissemination and poor outcome [1]. Synchronous adjacent DCIS and IBC foci show similar transcriptomic and genomic profiles, and few progression markers have been identified so far [2, 3]. Thus, a better understanding of the mechanisms and players underlying the progression to the invasive disease is needed in order to improve treatment decision and outcome.

Nucleoside diphosphate kinases (NDPKs), the products of the evolutionary conserved *NME/NM23* gene family, catalyze phosphate transfer from nucleoside triphosphates (mostly ATP) to nucleoside diphosphates [4]. Interestingly, NME1/NM23-H1 has been identified as the first metastasis suppressor, showing reduced expression in highly melanoma metastatic cells and as a suppressor of breast, liver, and colon carcinoma metastasis [5, 6]. At the mechanistic level, mutations in the drosophila *nme* homolog, *abnormal wing discs* (*awd*), are associated with developmental defects, and genetic studies have linked awd with *shibire* (*shi*), the gene encoding dynamin GTPase, required for membrane fission in the endocytic pathway [7]. We recently reported that human NME1 interacts with and supplies dynamin with high GTP levels required for membrane fission and, consequently, promotes endocytosis and clearance of cell surface receptors [8, 9].

A hallmark of metastasis is the acquisition of an invasive program enabling cancer cells to remodel the extracellular matrix (ECM) and disseminate. MT1-MMP (aka MMP-14), a trans-membrane matrix metalloproteinase, is required for DCIS-to-IBC progression and local invasion in the mammary gland. In addition, MT1-MMP up-regulation has been associated with higher metastatic risk in breast cancer [10–13]. It is well established that MT1-MMP is essential for carcinoma cell invasion by allowing the pericellular degradation of basement membrane and collagen-rich interstitial tissue barriers by cancer cells [14, 15]. A balance of endocytic and exocytic fluxes is thought to ensure a constant supply of active MT1-MMP at the plasma membrane [15–19]. MT1-MMP can be efficiently internalized by clathrin-mediated endocytosis and it is essential to better understand the molecular mechanisms that control its clearance from the cell surface.

The metastasis-suppressor role of NME1 together with its promoting function of dynamin activity in endocytosis, as well as the regulation of MT1-MMP surface exposure through endocytosis raise the intriguing possibility of a control of MT1-MMP activity by NME1 NDPK in cancer cells. The potential implication of NME1 and its close relative NME2 protein, during the invasive DCIS-to-IBC switch during breast cancer progression has been overlooked. Here, we investigated the expression of NME1 and NME2 in synchronous DCIS and IBC foci in breast tumors. We found an up-regulation of NME1 in DCIS as compared to normal peritumoral breast tissues and further down-regulation in invasive disease components. NME2 expression, which was similarly up-regulated in DCIS, remained high in IBCs. The ability of NME1 or NME2 to influence tumor invasion was evaluated using the intraductal xenograft model involving the injection of human MCF10DCIS.com cells into the primary duct of mouse mammary glands [11, 20]. Suppression of NME1, but not that of NME2, accelerated the invasive switch of MCF10DCIS.com tumor xenografts in the intraductal injection model. Finally, we found a specific association of NME1 with endocytic clathrin-coated structures and a regulation of MT1-MMP surface levels by dynamin downstream of NME1, clarifying the mechanism underlying the increased invasive potential of breast cancer cells during the DCIS-to-IBC transition.

## Experimental procedures

### Cell culture

See SI Experimental Procedures.

### Materials

For DNA constructs, antibodies, production and purification of recombinant proteins, see SI Experimental Procedures.

### RNA interference and CRISPR/Cas9 technology

See SI Experimental Procedures and SI Table S1.

### Human breast tumor samples and tissue microarray construction

Approximately 160 samples of primary breast tumors harboring synchronous DCIS and IBC, and 37 microinvasive breast carcinomas were collected at Institut Curie (SI Table S2). Analysis of the human samples by immunohistochemistry was performed, as detailed in SI Experimental Procedures.

### Immunohistochemical (IHC) staining of breast tumor tissue microarray

IHC was performed using validated highly selective NME1 and NME2 polyclonal antibodies, as detailed in SI Experimental Procedures.

### Unsupervised hierarchical clustering

The membranous H-score of MT1-MMP and the total H-score of NME1 from in situ and infiltrating tumor samples were scaled and then analyzed by unsupervised hierarchical clustering, as detailed in SI Experimental Procedures.

### Intraductal transplantation method

The intraductal xenograft model was carried out as previously described [11, 20]. For details, see SI Experimental Procedures.

### Histological and immunofluorescence analysis of mouse tissue sections

See SI Experimental Procedures.

### 3D collagen I invasion assay, quantification of pericellular collagenolysis, multicellular spheroid outgrowth in 3D matrigel, analysis of MT1-MMP cell surface expression, MT1-MMP internalization

See SI Experimental Procedures.

### In situ proximity ligation assay (PLA)

See SI Experimental Procedures.

### Immunoprecipitation

See SI Experimental Procedures.

### Subcellular fractionation

See SI Experimental Procedures.

### Pull-down assay

See SI Experimental Procedures.

### Statistical analysis

See SI Experimental Procedures.

## Results

### Down-regulation of NME1 in microinvasive breast cancers and in IBCs

**Fig. 1:**
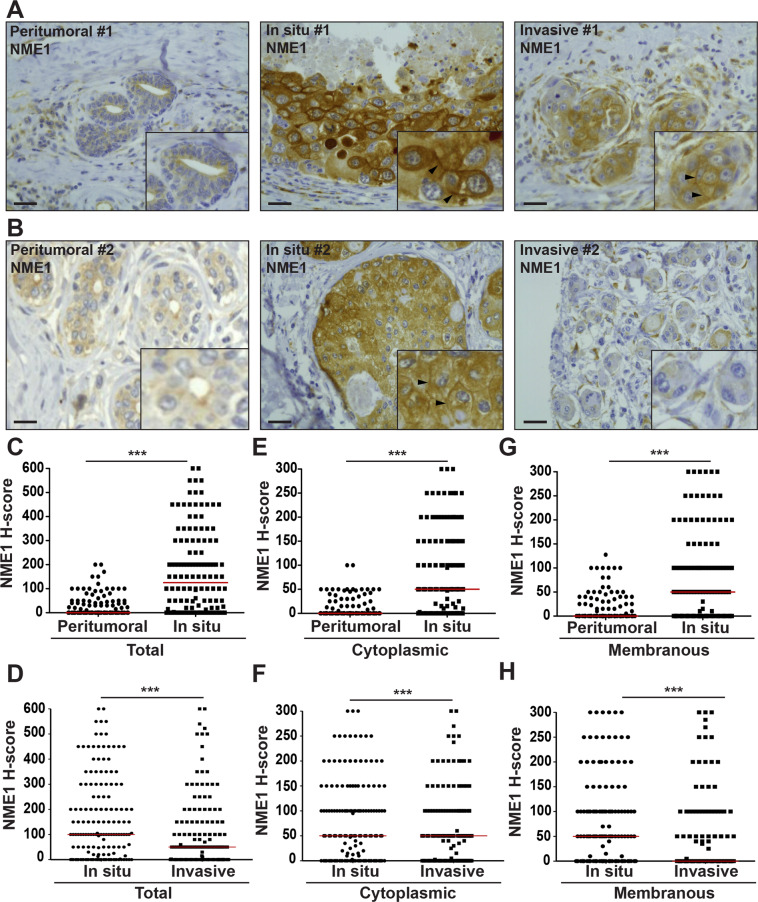
Biphasic up- and down-modulation of NME1 expression during breast cancer progression. **A**, **B** Representative NME1 IHC staining in breast peritumoral tissues and synchronous in situ and invasive components from two breast carcinoma biopsies. Arrowheads point to submembranous staining. Scale bar, 25 μm. **C**, **E**, **G** Comparison of total (**C**), cytoplasmic (**E**) and plasma membrane (**G**) NME1 levels using the H-score method in the in situ breast carcinomas as compared to adjacent breast peritumoral tissues. ***P < 0.001. **D**, **F**, **H** Total (**D**), cytoplasmic (**F**), and plasma membrane (**H**) NME1 levels were compared in synchronous in situ and invasive components of breast tumor biopsies. ***P < 0.001. The median of each H-score distribution is represented (red bar).

NME expression was investigated by IHC analysis on whole sections and on a tissue microarray (TMA) of synchronous DCIS and IBC foci from 156 breast cancer patient samples using specific NME1 pAb and NME2 mAb with no cross-reactivity (Supplementary Fig. S1 and Table S2). Contrasting with low or undetectable NME1 levels in healthy breast epithelial cells, NME1 was significantly up-regulated in DCIS cells, in which a strong cytoplasmic and peripheral staining was observed (Fig. 1A–C and E, G and Supplementary Fig. S2). In addition, NME1 levels were lower in IBCs relative to synchronous DCIS foci considering either total or separated cytoplasmic and membranous NME1 staining (Fig. 1D, F, H and Supplementary Fig. S2). When tumors were stratified into luminal A/B, HER2 + and triple-negative breast cancer (TNBC) subtypes, cytoplasmic and membranous NME1 levels remained significantly lower in the invasive component as compared to the adjacent DCIS foci, irrespective of the molecular subtype (Supplementary Fig. S3A–C). In addition, NME1 expression was lower in TNBC relative to luminal tumors, the former being aggressive and poor outcome tumors, irrespective of the in situ or invasive contingents (Supplementary Fig. S3D, E). Identical results were obtained using mouse NME1 mAb (Supplementary Fig. S1A and Fig. S4). Strikingly, NME1 level was also strongly decreased in microinvasive foci (a relatively rare tumor subset corresponding to early loco-regional invasion with no invasive focus > 1 mm [21, 22]), relative to the in situ component from the same specimen (Supplementary Fig. S5). Collectively, these findings indicate that down-regulation of NME1 NDPK correlates with the onset of breast cancer invasion.

### NME2 up-regulation during breast cancer progression

NME2 staining using a validated, highly selective mAb (Supplementary Fig. S1B–D) was visible at the apical surface of luminal epithelial cells in normal breast tissues (Supplementary Fig. S6A, B, left row, arrows). Similar to NME1, NME2 cytoplasmic staining was strongly up-regulated in DCIS as compared to adjacent normal cells (Supplementary Fig. S6A, B, middle row and Fig. S6C, E), while there was no difference considering the plasma membrane association of NME2 (Supplementary Fig. S6G). However, in sharp contrast to the drop in NME1 expression in IBCs, NME2 remained elevated in IBCs similar to its levels in DCIS (Supplementary Fig. S6A, B, right row and Fig. S6D, F, H).

### Anti-correlation of NME1 and MT1-MMP in breast cancer cells

**Fig. 2:**
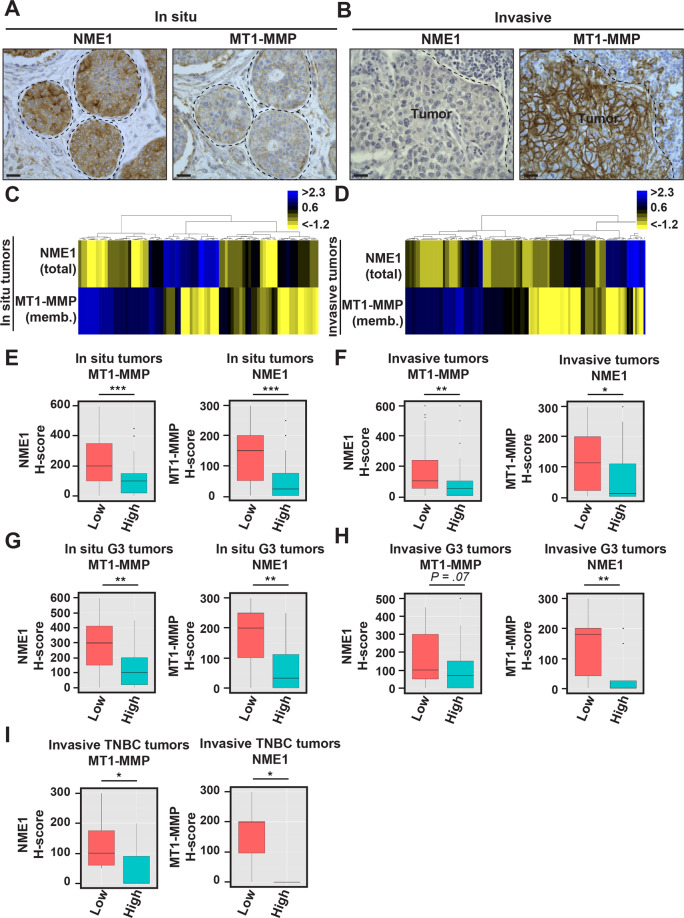
Anti-correlation of NME1 and MT1-MMP cell surface levels in human breast tumors. **A**, **B** Representative immunostaining of NME1 and MT1-MMP on serial sections of synchronous in situ (**A**) and invasive (**B**) components of human breast carcinoma (case #1). Scale bar, 25 μm. **C**, **D** Unsupervised hierarchical clustering based on total NME1 and plasma membrane MT1-MMP expression levels in the in situ (**C**) and invasive (**D**) breast carcinoma samples. Data are shown in a table format with the vertical axis listing the biopsies. A color scale, which represents the relative staining patterns of each sample, is displayed at the top right corner. **E**, **F** Left, box-plots of NME1 protein levels (H-score) depending on membranous MT1-MMP H-score variable discretized as low and high expression from in situ (**E**) and invasive components (**F**) of the cohort of human breast tumors. Right, box-plots of membranous expression of MT1-MMP (H-score) depending on NME1 H-score variable discretized as low and high levels in in situ (**E**) and invasive components (**F**) of the human breast tumor cohort. ***P < 0.001; **P < 0.01; *P < 0.05. **G**, **H** Box-plots of NME1 (left) or membranous MT1-MMP levels (right) depending on the reciprocal marker segregated as low and high expression from in situ (**G**) or invasive components (**H**) of higher histological grade III (G3) breast tumors. **P < 0.01. **I** Box-plots of NME1 (left) or membranous MT1-MMP levels (right) depending on the reciprocal marker segregated as low and high expression from invasive TNBC tumors. *P < 0.05.

Given the up-regulation of MT1-MMP during the in situ-to-invasive transition in relation with poor clinical outcome [11], we compared NME1 and MT1-MMP levels in the breast tumor cohort. We observed a striking anti-correlation of cortical MT1-MMP and NME1 both in DCIS and IBC breast tumor counterparts (Fig. 2A, B and Supplementary Fig. S7). NME1 and cortical MT1-MMP levels were scaled (Supplementary Fig. S8) and analyzed by unsupervised hierarchical clustering method, confirming the strong anti-correlation between the two proteins both in DCIS and IBC contingents (Fig. 2C–F). Anti-correlation was observed in high histological grade III as compared to lower-grade tumors (Fig. 2G, H) and in invasive TNBC tumors (Fig. 2I). All together, our data uncovered a biphasic NME1 alteration in breast cancer with a characteristic up-regulation in DCIS lesions and a robust down-modulation at the onset of the invasive switch and in invasive lesions. Down-regulation of NME1 correlated with the up-regulation of the pro-invasive, pro-metastatic surface MT1-MMP in IBCs.

### Acceleration of the invasive switch by loss of NME1 in the intraductal xenograft model

**Fig. 3:**
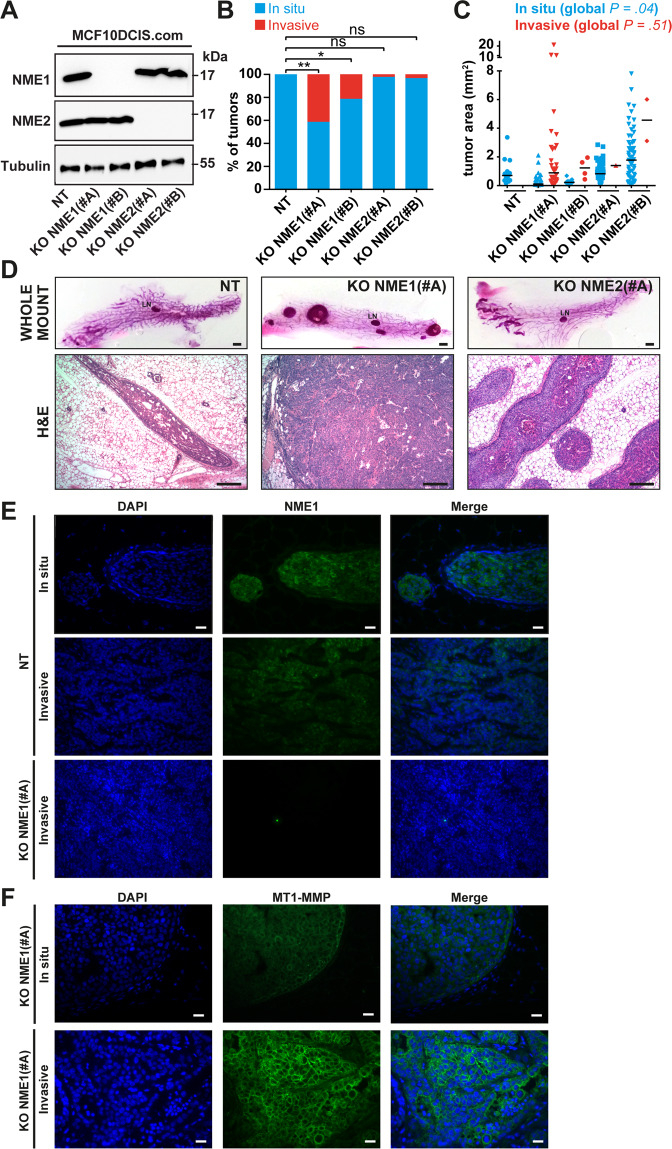
Loss of NME1 function promotes the in situ-to-invasive breast tumor transition. **A** Lysates of MCF10DCIS.com clones knockout for NME1 or NME2, or control non-KO cells (NT) were analyzed by immunoblotting with the indicated antibodies. Alpha-tubulin was used as a loading control. Molecular weights are in kDa. **B** Phenotypic analysis of intraductal xenograft tumors of MCF10DCIS.com cell clones ablated for NME1 or NME2 measured 4 weeks post-intraductal injection (p.i.i.) based on whole-mount staining of the mammary glands. **P < 0.01; *P < 0.05; *ns* not significant. **C** Tumor area of intraductal xenograft individual tumors of NT (wild type NME1 and NME2), KO NME1, or KO NME2 MCF10DCIS.com cells 4 weeks p.i.i. after phenotypic classification into in situ or invasive status. **D** Whole-mount carmine (upper row) and H&E staining (lower row) of nipple-injected glands 4 weeks after injection of the indicated MCF10DCIS.com cell populations. Scale bars, 1 mm (whole-mount carmine), 50 μm (H&E). **E** DAPI (blue) and NME1 (green) immunofluorescence staining of sections of in situ (upper row) or invasive (lower row) intraductal tumor xenografts of control NT MCF10DCIS.com cells 4 weeks or 7 weeks after intra-nipple injection, respectively. Scale bar, 40 μm. **F** DAPI (blue) and MT1-MMP (green) immunofluorescence staining of intraductal tumor xenografts of MCF10DCIS.com cells knockout for NME1 at the in situ (upper row) or invasive (lower row) stage. Scale bar, 20 μm.

Intra-nipple injection of human breast carcinoma MCF10DCIS.com cells in the mammary glands of SCID mice generate intraductal tumors that recapitulate the DCIS-to-IBC transition [20, 23]. Using this model, we reported that loss of MT1-MMP function impaired the invasive switch [11]. The consequences of the CRISPR/Cas9-mediated knockout of NME1 or NME2 were investigated using the intraductal xenograft model (Fig. 3A and Supplementary Fig. S9). Immunoblotting analysis showed that the knockout of NME1 did not affect NME2 levels and reciprocally (Fig. 3A). In addition, immunohistochemistry staining confirmed the loss of NME1 expression in tumor xenografts generated from NME1 knockout cells (Fig. 3E, lower panel KO NME1#A). At an early time-point after intraductal injection (i.e., 4 weeks), the tumor foci generated by control MCF10DCIS.com (NT) cells were all scored in situ based on histological staining of whole-mount and tissue-sections (Fig. 3B, D). Similarly, NME2 KO cells gave rise mostly to in situ tumors, with a very small subset of tumor foci (2–3%) having invasive features (Fig. 3B, D). In contrast, intraductal injection of NME1 KO cells generated up-to 20–40% of invasive tumors (Fig. 3B, D). Some size differences were also found with larger tumors obtained upon injection of NME2 KO cells (Fig. 3C, D), which correlated with higher percentage of PCNA-positive cells in NME2-KO tumor xenografts as compared to NT or NME1-KO tumors (Supplementary Fig. S10). Therefore, overgrowth of NME2-negative tumors was related to an increase in the proliferation rate but not to the invasive status.

Strikingly, at later time-point (i.e. 7 weeks after injection), immunostaining revealed that invasive tumor xenografts obtained upon injection of control MCF10DCIS.com cells expressed low NME1 levels, in contrast to in situ tumors that were frankly positive (Fig. 3E). In addition, NME1 knockout correlated with a strong membranous MT1-MMP expression in carcinoma cells that increased in IBC vs. DCIS tumors (Fig. 3F). Therefore, the invasive switch in xenograft tumors recapitulated the main features described in human breast tumors. Collectively, these data indicated that the loss of NME1, but not of NME2, accelerated the invasive switch of breast tumors in the intraductal xenograft assay, possibly in relation with increased plasma membrane MT1-MMP levels. Thus, loss of NME1 in breast carcinoma cells is a key emerging feature of the in situ-to-invasive breast carcinoma transition.

### NME1 controls the endocytic clearance and surface levels of MT1-MMP

**Fig. 4:**
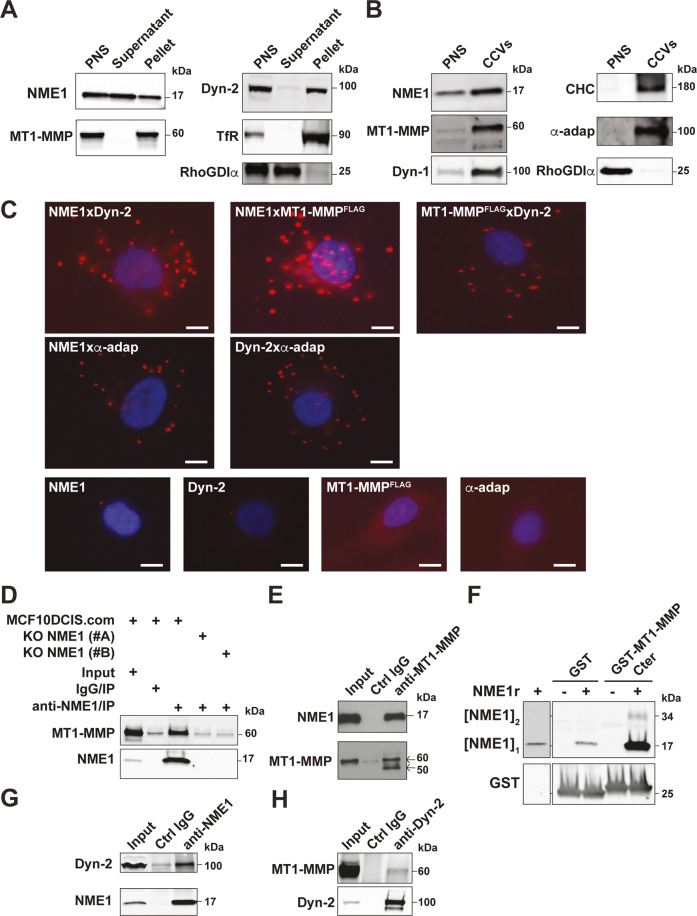
NME1 interacts with MT1-MMP and dynamin-2 in clathrin-coated vesicles. **A** After homogenization, a post-nuclear supernatant (PNS) of MCF10DCIS.com cells was ultracentrifuged to produce soluble (Supernatant) and membrane (Pellet) fractions. Proteins corresponding to equivalent cell-number were loaded in each lane and analyzed by immunoblotting with the indicated antibodies. The transferrin receptor (TfR) was recovered in the membrane pellet fraction, while cytosolic RhoGDIα was enriched in the supernatant. Dyn-2, dynamin-2. **B** PNS and clathrin-coated vesicle (CCV) fractions (10 μg) isolated from porcine brain were analyzed by immunoblotting with the indicated antibodies. Data are representative of two independent fractionation experiments. CHC, clathrin heavy chain, α-adap, α-adaptin, Dyn-1, dynamin-1. **C** Proximity-Ligation Assay (PLA) in MCF10DCIS.com cells using the indicated antibody combinations. Lower row, background PLA signal in the presence of single primary antibody. Scale bar, 5 μm. **D** Lysates of MCF10DCIS.com cells or cells knocked out for NME1 (clones #A and #B) were immunoprecipitated with NME1 antibodies or control IgGs followed by immunoblotting analysis with MT1-MMP antibodies. 1% of total lysate was loaded as a control (input). **E** In reciprocal experiments, lysates were immunoprecipitated with MT1-MMP antibodies followed by immunoblotting analysis with NME1 antibodies. 1% of total lysate was loaded as a control (input). **F** Direct interaction between purified recombinant NME1 (NME1r) and the carboxy-terminal tail of MT1-MMP fused with GST (GST-MT1-MMP-Cter). GST is used as a control. Proteins were analyzed by immunoblotting with NME1 or GST antibodies as indicated. [NME1]_1_, monomer; [NME1]_2_, denaturation-resistant dimer. Lane 1 is a longer exposure of the NME1 immunoblot. **G**, **H** MCF10DCIS.com cell lysates were immunoprecipitated with NME1 (G) or dynamin-2 (H) antibodies and bound proteins were analyzed by immunoblotting with dynamin-2 or MT1-MMP antibodies as indicated. 1% of total lysate was loaded as a control (input).

In order to explore the mechanism underlying the enhancement of breast tumor invasion by NME1 down-modulation, the cellular distribution of NME1 was examined. In agreement with the membranous NME1 staining in IHC analyses (see above), NME1 was recovered in the dynamin-2-positive membrane fraction also enriched for MT1-MMP (Fig. 4A). Some association of NME1 with the cytosolic fraction was also detected (Fig. 4A). Furthermore, we found that NME1 as well as MT1-MMP, were enriched in a clathrin-coated vesicle fraction positive for clathrin heavy chain and in the α-adaptin subunit of the clathrin adaptor complex, AP-2 (Fig. 4B).

Proximity ligation assay (PLA) confirmed a close proximity of NME1 with dynamin-2 and α-adaptin [8] (Fig. 4C). In addition, we detected a close proximity between FLAG-tagged MT1-MMP and NME1 and dynamin-2 (Fig. 4C). Omission of any one of the primary antibodies abolished PLA signal (Fig. 4C). Therefore, our data identified a close proximity between NME1, α-adaptin, dynamin-2 and MT1-MMP in the clathrin-mediated endocytic pathway in agreement with previous findings implicating clathrin-mediated endocytosis in the internalization of MT1-MMP [16, 17, 24]. Furthermore, NME1 and MT1-MMP could be co-immunoprecipitated, and co-immunoprecipitation was abolished by NME1 knockout demonstrating specificity (Fig. 4D, E). Moreover, we found a direct interaction between recombinant NME1 and the carboxy-terminal tail of MT1-MMP fused with GST (Fig. 4F). In addition, NME1 and dynamin-2 were co-immunoprecipitated under similar conditions (Fig. 4G, H). All together, these data indicated that NME1 interacted with MT1-MMP and dynamin-2 in clathrin-coated pits.

**Fig. 5:**
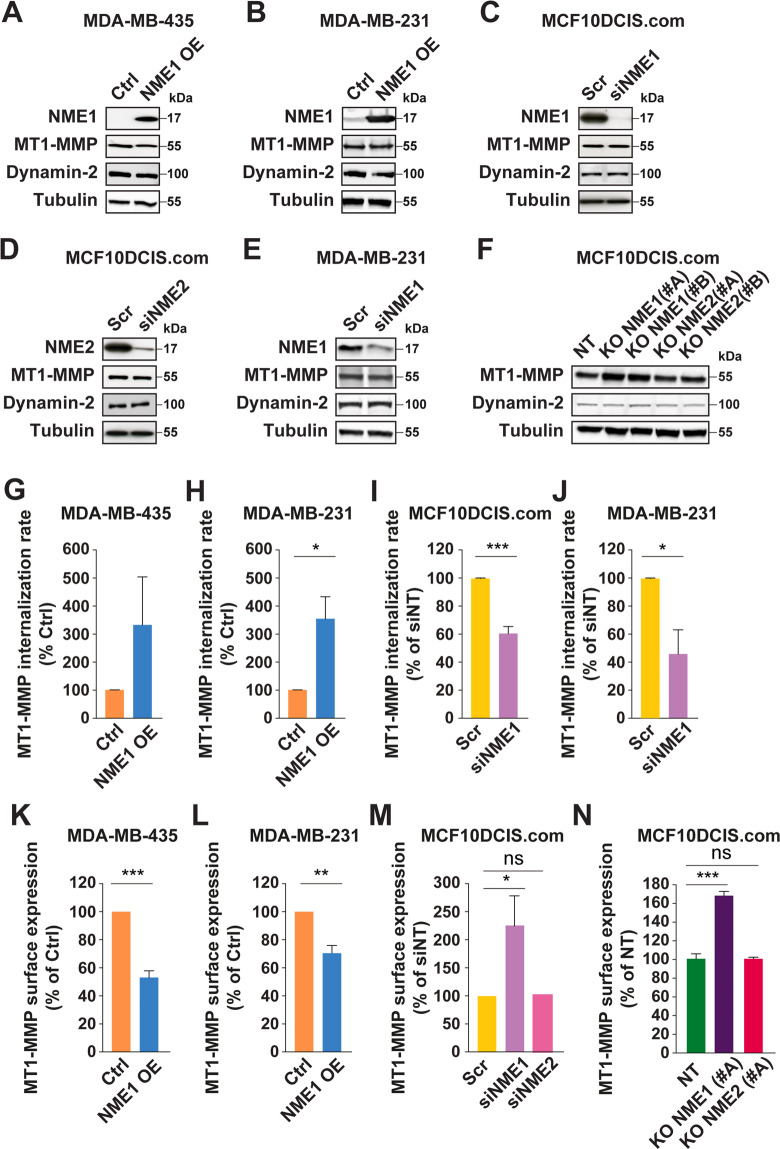
Modulation of NME1 levels impacts the endocytosis rate and surface levels of MT1-MMP in breast cancer cells. **A**, **B** Lysates of MDA-MB-435 and MDA-MB-231 cells overexpressing NME1 (NME1 OE) were analyzed by immunoblotting with the indicated antibodies. **C**, **D**, **E** MCF10DCIS.com or MDA-MB-231 cells were silenced for NME1 or NME2 by siRNA treatment and lysates were analyzed by immunoblotting with the indicated antibodies. **F** Lysates of MCF10DCIS.com cells knockout for NME1 or NME2, or control non-KO cells were analyzed by immunoblotting with the indicated antibodies. **G**, **H**, **I**, **J** NME1 levels were modulated by overexpression (**G**, **H**) or silencing (**I**, **J**) in the indicated breast cancer cell lines, and MT1-MMP endocytosis rate was measured using a cell-surface biotinylation assay after 60 min incubation at 37 °C. Three independent experiments were performed. Error bars are the standard error of the mean (SEM). ***P < 0.001; *P < 0.05. For (**G**), although not statistically significant, overexpression of NME1 in MDA-MB-435 cells clearly tended to increase MT1-MMP endocytosis (6.7-, 2.1-, and 1.2-fold increase for each of the three independent experiments). **K**, **L**, **M**, **N** NME1 levels were modulated in different breast cancer cell lines as indicated, and surface MT1-MMP levels were analyzed by FACS. Four independent experiments were performed for (**K**, **L**, **M**). Three independent experiments were performed for (**N**). Error bars are the standard error of the mean (SEM). ***P < 0.001; **P < 0.01; *P < 0.05; *ns* not significant.

We previously identified NME1 as an enhancer of dynamin GTPase-mediated endocytosis by providing GTP supply for dynamin’s proper function in vesicle sission [8]. Therefore, we investigated the impact of genetically modified NME1 levels on the rate of MT1-MMP internalization in human breast cancer cell lines. Overexpression of NME1 in MDA-MB-435 and MDA-MB-231 cells significantly increased MT1-MMP endocytosis (Fig. 5A, B, G, H), whereas silencing of NME1 in MCF10DCIS.com or MDA-MB-231 cells significantly decreased MT1-MMP uptake (Fig. 5C, E, I, J).

Surface-exposed MT1-MMP results from a balance of endocytic and exocytic events, and is responsible for pericellular degradation of ECM components by carcinoma cells [15, 18, 19]. Immunoblot analysis in breast cancer cell lines with genetically-modified NME1 or NME2 levels revealed no gross alteration of total MT1-MMP levels (Fig. 5A–F). Surface MT1-MMP levels were analyzed using a validated flow cytometry assay (Supplementary Fig. S11). Overexpression of NME1 in MDA-MB-435 or MDA-MB-231 cells resulted in a strong reduction of surface MT1-MMP levels (Fig. 5K, L). In reciprocal experiments, loss of NME1 function in MCF10DCIS.com cells resulted in a ~2-fold increase in MT1-MMP surface expression, while loss of NME2 had no such effect (Fig. 5C, D, F, M, N). Collectively, these data indicate that metastasis-suppressor NME1, but not NME2, controls the endocytic clearance and surface exposure of MT1-MMP in various breast cancer cell lines.

### NME1 regulates MT1-MMP-dependent pericellular collagenolysis and invasion

**Fig. 6:**
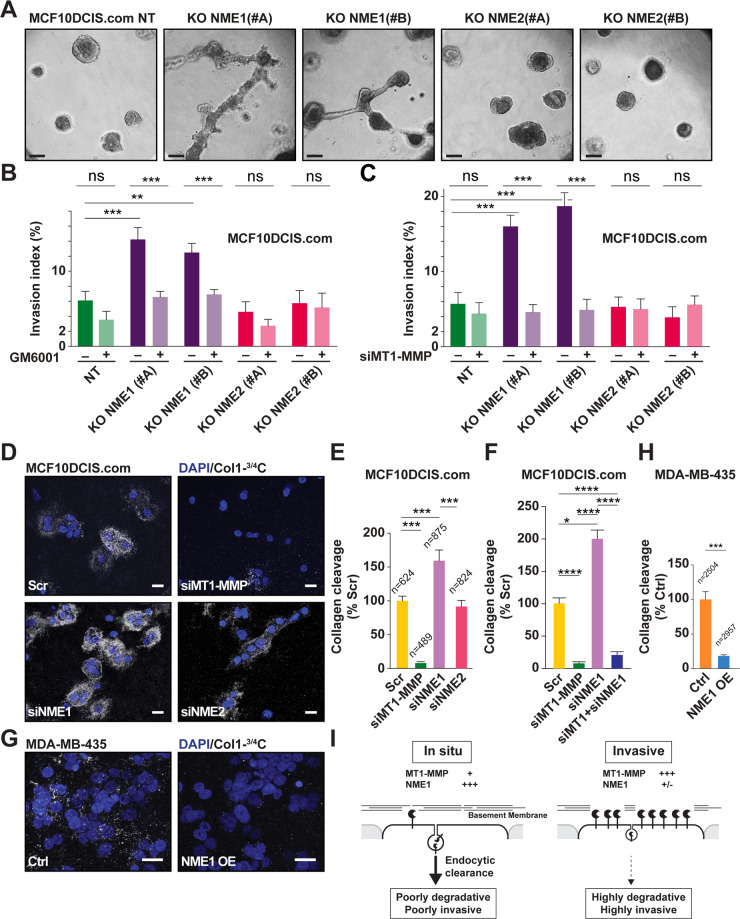
NME1 controls invasive and matrix-degradative potential of breast cancer cells through MT1-MMP activity. **A** Independent clones of MCF10DCIS.com cells knockout for NME1 or NME2 or non-KO control cells (NT) were embedded in Matrigel as a single cell suspension followed by a 7-d culture and imaged by differential interference contrast microscopy. Scale bar, 50 μm. **B** MCF10DCIS.com cells knockout for NME1 or NME2 were seeded on a type I collagen gel and cultured for 24 h in the absence or the presence of MMP inhibitor, GM6001. Invasive index represents the percentage of cells invading the gel divided by the total number of cells from two independent experiments. Error bars are the standard error of the mean (SEM). ***P < 0.001; **P < 0.01; *ns* not significant. **C** MCF10DCIS.com cells knockout for NME1 or NME2 were silenced for MT1-MMP expression by siRNA treatment and assayed for type I collagen invasion as in panel (**B**). Two independent experiments were performed. Error bars are the standard error of the mean (SEM). ***P < 0.001; *ns* not significant. **D** MCF10DCIS.com cells treated with the indicated siRNAs were embedded in collagen I and stained for cleaved collagen I with Col1-^3/4^C pAb (white). DAPI-stained nuclei are shown in blue. Scale bar, 20 μm. **E** Pericellular collagenolysis was detected using Col1-^3/4^C pAb as in panel (**D**). Values are mean normalized degradation index ± SEM. *n* number of cells analyzed for each cell population from three independent experiments. ***P < 0.001. **F** MCF10DCIS.com cells treated with the indicated siRNAs were embedded in collagen I and pericellular collagenolysis was scored as in panel (**D**, **E**). **G**, **H** MDA-MB-435 cells transfected with the empty control vector (Ctrl) or overexpressing NME1 (NME1 OE) were embedded in type I collagen and pericellular collagenolysis was detected and scored. Scale bar, 20 μm. **I** Scheme recapitulating the function of NME1 in promoting the endocytic clearance of MT1-MMP in relation with the invasive switch of breast cancer.

We assessed the contribution of NME1 to the capacity of tumor cells to remodel and invade through matrix constructs consisting either of Matrigel, with a composition similar to the basement membrane, or type I collagen, the main component of the interstitial tissue. While, control and NME2-KO MCF10DCIS.com cells grew as compact spheroids in Matrigel, spheroids of cells KO for NME1 formed invasive outgrowths (Fig. 6A). Induction of an invasive program by loss of NME1 was similarly observed in cells embedded in the 3D type I collagen network, and was abolished in the presence of GM6001, a general MMP inhibitor, or upon silencing of MT1-MMP indicating that the invasion program induced by NME1 loss-of-function required MT1-MMP activity (Fig. 6B, C and Supplementary Fig. S12A).

Finally, we investigated the consequences of the modulation of NME1/NME2 levels on the ability of tumor cells to proteolytically cleave the surrounding type I collagen fibers. We used the Col1-^3/4^C mAb, which recognizes MMP-cleaved type I collagen molecules [25, 26]. Silencing of MT1-MMP in MCF10DCIS.com cells abolished pericellular collagenolysis indicating that type I collagen degradation strongly relied on MT1-MMP activity (Fig. 6D, E). Knockdown of NME1 led to a 1.5-2-fold increase in pericellular collagenolysis, in contrast to NME2 silencing that did not affect collagenolysis (Fig. 6D–F and Supplementary Fig. 12B). Moreover, silencing of MT1-MMP abolished the induction of pericellular collagenolysis upon NME1 loss of function (Fig. 6F). Collectively, these data indicate that MT1-MMP mediates both an increase in invasion and collagenolysis in breast cancer cells with reduced NME1 activity.

The regulation of collagenolysis by NME1 could be generalized by overexpression in MDA-MB-435 cells that express barely detectable level of NME1 (Fig. 5A), resulting in a strong 80% reduction of collagenolysis (Fig. 6G, H). Thus, we conclude that NME1, not NME2, is an essential element of the pericellular matrix proteolysis and invasion programs of breast tumor cells by controlling the clearance and surface expression of MT1-MMP in breast carcinoma cells (see Model in Fig. 6I).

## Discussion

Down-modulation of NME1 NDPK expression is known to correlate with metastatic dissemination and worse prognosis in several cancer types, including breast cancers [27–29]. However, NME1’s implication in local invasion at the in situ-to-invasive breast carcinoma transition has been overlooked, and the mechanisms underlying metastasis suppression by NME1 remained largely unknown.

Our IHC analysis of breast tumor specimens based on highly discriminating antibodies revealed an up-regulation of NME1 levels in carcinoma cells in DCIS tumors as compared to surrounding non-malignant tissues, whereas NME1 levels were significantly reduced in synchronous invasive tumor foci and in microinvasive carcinoma buds extending beyond the ruptured basement membrane. Thus, we propose NME1 as a potential marker to predict in situ tumors with high risk to progress into invasive breast carcinomatous lesions, which remains a critical issue in breast cancer management [30]. Supporting our conclusion, NME1 is also reduced or absent at the invasive front of human hepatocellular carcinoma and colon cancers, as compared to its strong expression in the tumor central area [31]. Taken together, these data suggest that the reduction of NME1 expression during the progression to invasive disease is a generic feature of epithelial tumor progression.

We found a strong anti-correlation of NME1 and cortical MT1-MMP expression in invasive breast carcinomatous lesions. Anti-correlation of NME1/MT1-MMP levels was confirmed in RNAseq data in invasive breast carcinoma (TCGA, not shown). This anti-correlation was additionally observed in carcinomas of various origin including colon, endometrial, ovarian, prostate, and head and neck squamous carcinoma tumors (TCGA, not shown), indicating that negative control of MT1-MMP activity in matrix remodeling is a generic trait of the metastasis-suppressive function of NME1, which is lost upon repression of NME1 expression during cancer progression. In addition, based on the intraductal xenograft model, [20], we have shown that down-modulation of NME1 accelerated the invasive transition in breast carcinoma. Together with the potent inhibition of the invasive switch caused by the loss of MT1-MMP in the intraductal model [11], these convergent findings suggested that the loss of NME1 function in breast tumor epithelial cells could unleash MT1-MMP proinvasive activity. Complex regulatory networks based on transcriptional regulators including p63 and AIB1/YAP have been identified as drivers of malignant progression, invasion and proliferation during breast cancer progression [2, 11, 32, 33]. Whether these different regulatory circuits play a role in the biphasic NME1 expression profile and MT1-MMP up-regulation during breast cancer progression will be interesting to examine in future studies.

In order to work efficiently, dynamin, which has a low affinity for GTP and a high intrinsic GTPase activity, needs to be permanently reloaded with GTP, which is provided by NME1 and NME2 NDPKs [8]. In addition, NME1 has been proposed to facilitate the oligomerization and GTPase activity of dynamin [34]. Both GTP channeling and stimulation of dynamin oligomerization by NME1/2 contribute to the stimulation of dynamin function in vesicle scission. Endocytosis is a major mean by which cells regulate MT1-MMP cell surface levels, which directly impinge on pericellular ECM degradation [15, 18]. We show here that NME1 interacts directly with the cytosolic domain of trans-membrane MT1-MMP and with ubiquitously-expressed dynamin-2 in clathrin-coated pits, and that the reduction of NME1 function impairs both MT1-MMP endocytosis in relation with an increase in surface exposure, and consequent enhancement of the degradation of the pericellular ECM mediated by MT1-MMP (see Model in Fig. 6I). These data are in agreement with a marked defect in internalization and strong enhancement of collagenolysis reported for a tail-deleted MT1-MMP construct [24]. Noteacibly, we and others reported that, besides its role in endocytosis at the plasma membrane, dynamin-2 also localizes to (MT1-MMP-positive) endolysosomal compartments where it is required for the recycling of MT1-MMP and co-trafficking cargoes, such as flotillins, from endolysosomes back to the surface [35–38]. Inhibition or silencing of dynamin-2 was shown to impair invadopodia activity and matrix degradation [36], opposite of what is seen upon NME1 inhibition. Furthermore, dynamin-2 has been shown to act as a positive regulatory factor of matrix degradation and metastasis, in relation with its role in actin cytoskeleton organization and actin dynamics at invadopodia and in podosomes [39–42]. Therefore, the regulation of ECM degradation by dynamin-2 is complex and not limited to dynamin-2’s role in MT1-MMP surface clearance through clathrin-mediated endocytosis.

A further layer of complexity is that the modulation of MT1-MMP surface levels, in relation with changes in NME1 expression such as the one we found in breast cancers, may affect other MMPs’ function and membrane proteins that are known to be shedded by MT1-MMP, such as integrins or CD44, also with consequence for invasion and metastasis [15]. In preliminary analyses, we found that similar to NME1, NME2 can be detected in membrane and cytosolic fractions prepared from MCF10DCIS.com cells, and is enriched in a clathrin-coated vesicle fraction (Supplementary Fig. S13A, B). This distribution is expected given that NME2 interacts and forms catalytically active hetero-hexamers with NME1 [4, 8]. In addition, NME2 could be co-immunoprecipitated with MT1-MMP (Supplementary Fig. S13C). At this stage, information regarding the relative expression of NME1 and NME2 and stoichiometry of NME1/NME2 hetero-hexamers in different breast cancer cells is missing. In addition, we do not know whether co-immunoprecipitation of NME2 with MT1-MMP relies on a direct interaction between these two proteins or is mediated by another protein that could be NME1. Yet, our data clearly support the conclusion that NME2 knockout, that does not affect NME1 expression levels (Fig. 3A), does not impair the invasive and collagenolysis capacity of MCF10DCIS.com cells, in sharp contrast with NME1 loss of function (Fig. 6A–E).

Here, we report a role for NME1 on the acquisition of invasive traits in breast epithelial cancer cells. As the loss of NME1 may be a prerequisite for the induction of invasive features in patients with DCIS, we anticipate that its clinical management may prevent or delay the invasive switch of breast cancers. In this regard, NME1 expression in breast cancer as well as other carcinomas might be used as a prognostic factor for monitoring progression to invasive states. Finally, while therapeutic efforts aimed at targeting a proteolytic enzyme that undergoes continuous recycling at the cell surface might prove problematic, therapies directed at increasing NME1 expression might prove effective at preventing or interfering with the tissue-invasive behavior of aggressive breast cancers.

## Supplementary information

Supplemental material
