# Supplementary material for: Metastasis-suppressor NME1 controls the invasive switch of breast cancer by regulating MT1-MMP surface clearance
Source: Oncogene. 2021 May 19;40(23):4019–32. doi: 10.1038/s41388-021-01826-1 (PMC8195739; doi:10.1038/s41388-021-01826-1)

Metastasis-suppressor NME1 controls the invasive switch of breast cancer by regulating MT1-MMP surface clearance

Catalina Lodillinsky[1,2], Laetitia Fuhrmann[3], Marie Irondelle[4], Olena Pylypenko[4], Xiao-Yan Li[5], Hélène Bonsang-Kitzis[6,7,¶], Fabien Reyal[6,7], Sophie Vacher[8], Claire Calmel[9], Olivier De Wever[10], Ivan Bièche[8], Marie-Lise Lacombe[9], Ana Maria Eiján[1,2], Anne Houdusse[4], Anne Vincent-Salomon[3], Stephen J. Weiss[5], Philippe Chavrier[4,‡] and Mathieu Boissan[9,11,‡,*]

[‡] Philippe Chavrier and Mathieu Boissan contributed equally to the work

[1] Research Area, Instituto de Oncología Ángel H. Roffo, Universidad de Buenos Aires, Buenos Aires, Argentina

[2] Consejo Nacional de Investigaciones Científicas y Técnicas (CONICET), Buenos Aires, Argentina

[3] Institut Curie, PSL Research University, Diagnostic and Theranostic Medicine Division, Paris, France

[4] Institut Curie, PSL Research University, CNRS UMR144, Paris, France

[5] Division of Medical Genetics, Department of Internal Medicine, Life Sciences Institute, University of Michigan, Ann Arbor MI, USA

[6] Department of Surgery, Institut Curie, Paris, France

[7] Translational Research Department, RT2Lab Team, INSERM U932, Immunity and Cancer, Institut Curie, Paris, France

[8] Pharmacogenomic Unit, Institut Curie, Paris, France

[9] University Sorbonne, INSERM UMR_S 938, Saint-Antoine Research Center, CRSA, Paris, France

[10] Laboratory of Experimental Cancer Research, Cancer Research Institute Ghent (CRIG), Department of Human Structure and Repair, Ghent University, Ghent, Belgium

[11] Laboratory of Biochemistry and Hormonology, Tenon Hospital, AP-HP, Paris, France

¶ Present address: Gynecological and Breast Surgery and Cancerology Center, RAMSAY-Générale de Santé, Hôpital Privé des Peupliers, Paris, France

* Corresponding author: Mathieu Boissan, University Sorbonne, INSERM UMR_S 938, Saint-Antoine Research Center, CRSA, Paris, France. E-mail: mathieu.boissan@inserm.fr; Tel: +33 1 49 28 46 32

Supplemental experimental procedures

Cell culture

MCF10DCIS.com cell line was purchased from Asterand (Detroit, MI, USA) and maintained in advanced DMEM/F12 media supplemented with 5% horse serum and 2 mM glutamine at 37°C in 5% CO_2. MDA-MB-435 were maintained in DMEM supplemented with 10% fetal calf serum and 2 mM glutamine at 37°C in 5% CO_2. MDA-MB-231 cell line was obtained from the American Type Culture Collection (ATCC HTB-26, LGC Promochem) and maintained in L-15 culture medium with 2 mM glutamine and 15% fetal calf serum at 37°C in 1% CO_2.

DNA constructs

C-terminally FLAG-tagged MT1-MMP construct was generated by inserting primers encoding the FLAG epitope (5'-GACTACAAGGACGACGATGACAAG-3') after the COOH-terminal valine of the molecule and cloned in pCR3.1 Uni as previously described [1].

Antibodies

Highly specific NME1 and NME2 rabbit polyclonal antibodies (pAbs) were previously described [2]. Rabbit pan-NME pAb (recognizing both NME1 and NME2 isoforms) were prepared by affinity purification using purified human recombinant NME1 and NME2 proteins coupled to NHS-activated HiTrap columns [2]. Mouse NME1 (UMAB94) and NME2 (clone KM1121) monoclonal antibodies (mAbs) were purchased from OriGene (Rockville, MD) and Kamiya Biomedical Company (Seattle, WA), respectively. Rabbit dynamin-2 pAb and mouse dynamin-1 mAb were a kind gift of Dr. P. De Camilli (Yale University School of Medicine, New Haven, CT). Mouse α-adaptin

mAb (clone AC1-M11) was obtained from Abcam (Cambridge, MA, USA). Rabbit α-adaptin 1/2 (M-300) and Rho GDIα (A-20) pAbs were purchased from Santa Cruz Biotechnology (Santa Cruz, CA). Mouse MT1-MMP mAb (clone LEM-2/15.8) was purchased from Millipore (Molsheim, France) and used for western blotting, immunofluorescence and immunoprecipitation analyses. Mouse MT1-MMP mAb (clone 2D7, a kind gift from C.L. Tomasetto, IGBMC, Illkirch, France [3]), was used for flow cytometry analysis. Mouse PCNA mAb was purchased from Cell Signaling Technology (Beverly, MA). Mouse transferrin receptor mAb (clone H 68.4) was obtained from Zymed Laboratories (South San Francisco, CA). Mouse α-tubulin mAb (clone DM1A) was purchased from Thermo Fisher Scientific (St. Louis, MO).

RNA interference

All siRNA oligonucleotides were synthesized by Ambion^{R} Life Technologies (Applied Biosystems, Austin, TX). The following siRNAs were used for NME1, pooled 5'-GGAUUCCGCCUUGUUGGUC-3' and 5'-GGCUGUAGGAAAUCUAGUU-3'; NME2, pooled 5'-GGAUUGAUCAUUCUUUUAU-3' and 5'-GCCUAUGGUUUAAGCCUGA-3'; irrelevant control siRNA: 5'-GGCUGUAGAAGCUAUAGUU-3'. MCF10DCIS.com cells were transfected with 100 nM control (scrambled siRNA) or specific siRNA duplex using Lullaby reagent (OZ Biosciences, Marseille, France). Protein depletion was verified by immunoblotting analysis with specific antibodies and was maximal after 72 hrs of siRNA treatment.

CRISPR/Cas9 technology

CRISPR guides. Lentiviral plasmid guides targeting human NME1 and NME2 were generated in pLenti U6gRNA Cas9-GFP-Puro vector and were purchased from Merck-

Sigma-Aldrich as well as the non-target guide (pLenti CRISPR-NT CONTROL). Two different guides were designed (https://www.milliporesigmabioinfo.com/bioinfo_tools/) for NME1: NME1-A [#HS0000009943, target sequence GACGGGCCGAGTCATGCTCGGG] and NME1-B [#HS0000009940, target sequence GAACACTACGTTGACCTGAAGG], and for NME2: NME2-A [#HS0000056847, target sequence TCATCGCCATCAAGCCGGACGG] and NME2-B [#NME2_0_76, target sequence AAGACCGACCATTCTTCCCTGG].

Lentiviral vectors production and MCF10DCIS.com cells transduction. These steps were performed with help of the GIGA Viral vectors platform (University of Liège, Belgium). Briefly, Lenti-X 293T cells (Clontech) were co-transfected together with pLenti U6gRNA NME1-Cas9-GFP-Puro or pLenti U6gRNA NME2-Cas9-GFP-Puro or pLenti CRISPR-NT CONTROL and with pcgpV (Cell Biolabs), pRSV-Rev (Cell Biolabs) and VSV-G (Cell Biolabs) encoding vectors. Lentiviral supernatants were collected 48 to 96 hrs post-transfection, filtrated and concentrated 100x by ultracentrifugation. Lentivirus stocks were titrated with qPCR Lentivirus Titration (Titer) Kit (abm) and used to transduce cells. After 72 hrs, cells were selected with 2 µg/ml puromycin (Cayla/Invivogen). Then, cells expressing GFP were isolated and cloned by FACS on a FACSaria III 4L sorter (BD Biosciences). Each clone was tested by western blotting and immunofluorescence analysis. Clones that were negative for NME1 or NME2 expression were selected for further experiments.

Sequencing. Selected clones were analyzed by miSeq in order to confirm mutations in NME1 or NME2-coding sequences. DNA were extracted from cell pellets using Maxwell® 16 Blood DNA Purification Kit. Primers flanking the CRISPR-Cas9 target sites were designed with Primer3 based on the UCSC hg19 human reference genome. Nextera XT adapter overhangs sequences and primers sequences are in Table S1.

For all clones, amplicons were generated for the four targeted regions (NME1A, NME1B, NME2A, and NME2B) using Q5 High-Fidelity DNA Polymerase (New England Biolabs). PCR1 products were purified with Ampure beads. Illumina sequencing adapters and dual index barcodes were added to the amplicon target libraries with only 8 cycles PCR using Kapa Hifi HotStart ready mix (as described in 16s workflow Illumina guide). Different combinations of Nextera XT index were used for each sample, PCR2 products were then purified with AMpure beads, quantified with picogreen dsDNA quantitation assay and normalized at 7 ng/µl and then pooled. Before proceeding to High throughput sequencing, the final pools were quantified by qPCR (KAPA SYBR FAST kit (ABI Prism). Final libraries were spiked (8%) into a Miseq run 300 cycles v2 (Read1: 156 cy, Read2:160 cy, index1: 8cy, index2: 8cy).

Sequence analysis. Raw reads were demultiplexed and adapter-trimmed using Illumina bcl2fastq. Analysis of the sequencing data was performed using CRISPResso v1.0.2 (Ref: https://www.ncbi.nlm.nih.gov/pubmed/27404874) comparing for each amplicon of each clone, the sequencing data to the corresponding region of the UCSC hg38 reference the Human genome. Reads containing insertions and/or deletion (indel) with respect to the reference amplicon sequence were identified and considered as edited while reads only containing substitutions were conservatively considered as not edited (CRISPResso options: --ignore_substitutions and no guide provided). The region of the amplicon containing coding sequences was also provided to identify out-of-frame indels (CRISPResso -c option). Analyses performed in CRISPResso with alternative options (counting modifications in a window of 7 nucleotides around the predicted cutting site and with or without ignoring substitutions) gave similar results. Sequencing results were also checked visually in the Integrative Genome Viewer after

alignment directly to the entire UCSC hg38 Human reference genome with BWA v0.7.5a (BWA mem algorithm).

Human breast tumor samples and tissue microarray construction

Approximately one hundred sixty samples of primary breast tumors harboring synchronous DCIS and IBC, and 37 microinvasive breast carcinomas (defined as infiltrating carcinomas with one or more areas of focal invasion, none larger than 1 mm in size) were collected at Institut Curie from 2005 to 2006 prior to any radiation, hormonal or chemotherapy treatment. Analysis of the human samples by immunohistochemistry (IHC) was performed in accordance with the French Bioethics Law 2004-800, the French National Institute of Cancer (INCa) Ethics Charter, and after approval by the Institut Curie review board and ethics committee (*Comité de Pilotage du Groupe Sein*). Patients were informed of the research use of their tissues and did not declare any opposition for such research. Tumors were classified as IBC, DCIS and microinvasive based on assessment by a pathologist [4]. Inclusion of DCIS and tumors followed same criteria as IBCs with additional marker assessment including Ki67 and p63. Microinvasive tumors were defined as infiltrating carcinomas with no invasive focus >1 mm. Tumor breast molecular subtypes were defined as follows according to the guidelines of the American Society of Clinical Oncology (ASCO)/College of American Pathologists [5, 6]: Luminal A: estrogen-receptor (ER)≥10%, progesterone-receptor (PR)≥20%, Ki-67<14%; Luminal B: ER≥10%, PR<20%, Ki-67≥14%; HER2+: ER<10%, PR<10%, HER2 2+ amplified or 3+; Triple-negative breast cancers (TNBC): ER<10%, PR<10%, HER2 0/1+ or 2+ non-amplified. The clinical and pathological features of patients are summarized in Table S2. The TMA consisted of arrayed 1-mm diameter cores from synchronous in situ and

microinvasive/invasive carcinomas and a matched core from adjacent non-tumoral breast tissue constructed as previously described [4].

Immunohistochemical (IHC) staining of breast tumor tissue microarray

Sections (3 µm) from paraffin-embedded tissue microarrays were dewaxed in xylene and rehydrated in a graded alcohol series before heat-induced antigen retrieval (60 minutes in 10 mM sodium citrate buffer, pH 6.0 at 90°C). Then, sections were incubated in 3% hydrogen peroxide solution for 5 min to inhibit endogenous peroxidase activity. The slides were further incubated with blocking serum for 15 min and then with the primary antibodies. Selective NME1 and NME2 pAbs were used: affinity-purified rabbit NME1 pAb [2] was used at a dilution of 1:3000; mouse NME2 mAb purchased from Kamiya Biomedical Company (Seattle, WA) was used at a dilution of 1:1000. Immunolabeling was performed using the Dako Autostainer Plus and EnVision™ FLEX, Low pH kit with diaminobenzidine as chromogen according to the manufacturer's procedure (Dako, Santa Clara, CA). Slides were counterstained with hematoxylin before mounting. Images were acquired with the Philips Ultra-Fast Scanner. NME1 and NME2 levels in the different tumor biopsies were scored under the supervision of a pathologist in a blinded manner using the H-score method based on semi-quantitative assessment of the intensity of plasma membrane and cytoplasmic staining (graded as: 0, non-staining; 1, weak; 2, median; or 3, strong) and the percentage of positive cells. H-score range was from 0 to 300 for membranous or cytoplasmic staining and 0 to 600 for total staining.

Unsupervised hierarchical clustering

The membranous H-score of MT1-MMP and the total H-score of NME1 from in situ

and infiltrating tumor samples were scaled and then analyzed by unsupervised hierarchical clustering using the Ward linkage clustering algorithm with Euclidean distance as the similarity metric (EMA package). Membranous MT1-MMP and total NME1 H-score variables were then discretized either in low or in high expression in order to perform association test. Cut-off values for H-scores were calculated using normal mixture modeling (Mclust R package). The same cut-off values were used for in situ and invasive components. Threshold membranous MT1-MMP: 125; threshold total NME1: 300. Membranous MT1-MMP and total NME1 H-scores were plotted as box-plot according to discretized membranous MT1-MMP and total NME1 H-scores, respectively, breast cancer subgroups, Elston and Ellis grade.

Intraductal transplantation method

The intraductal xenograft model was carried out as previously described [4, 7]. Briefly, $5x10^4$ MCF10DCIS.com cells in 2 μl PBS were injected into the primary duct through the nipples of both mammary inguinal glands #4 of 8-10 weeks-old virgin female SCID mice. Mice were sacrificed at 4 and 7 weeks after injection by cervical dislocation. Immediately after being euthanized, mammary glands were excised and processed for further study (including whole-mount and histological and IHC staining on sections). The animal facility was granted approval (C-75-12-01) given by the French Administration. All experiments were conducted according to the European Communities Council Directive (2010/63/UE) for the care and use of animals for experimental procedures and complied with the regulations of the French Ethics Committee in Animal Experiment 'Charles Darwin' registered at the *'Comité National de Réflexion Ethique sur l'Experimentation Animale'* (C2EA - 05). All procedures were approved by this committee (APAFIS#11806-2017101710125048 v2).

Histological and immunofluorescence analysis of mouse tissue sections

Whole-mount carmine and hematoxylin and eosin (H&E) staining of tissue sections were performed as described in [4]. After whole mount staining, image acquisition was performed with an A1R Nikon confocal binocular microscope. Quantification of the tumor area was performed using Image J software. To retrieve antigens on paraffin-embedded tissue samples, sections were incubated for 20 min in 10 mM sodium citrate buffer, pH 6.0 at 90°C. Then, after 60 min incubation in 5% fetal calf serum, sections were incubated overnight with diluted primary antibodies, washed and further incubated for 2 hrs at room temperature with appropriate secondary antibodies.

3D collagen I invasion assay

Details of the procedure have been described [8]. Briefly, Petri dishes were filled with 1.35 ml of neutralized native type I collagen and incubated overnight at 37°C to allow gelling. Cells were harvested and isolated using Moscona buffer and trypsin/EDTA, then seeded on the top of the collagen gel at the density of 0.33 $x10^6$ cells per dish. Cells were cultured for 24 hrs in the absence or presence of GM6001 (10 μM). Non-invasive round-shape cells that remained at the surface of the gel, and invasive cells that inserted an invasive extension within the collagen gel were scored in twelve fields of 0.157 mm^2. Invasion index was calculated as the number of cells with invasive extensions to the total cell number multiplied by 100 [8].

Quantification of pericellular collagenolysis

Cells treated with siRNAs against MT1-MMP, NME1, NME2, and non-targeting siRNA for 48 hrs, or stably overexpressing NME1 (or empty vector) were trypsinized,

resuspended (2.5 x 10^5 cells/ml) in 0.2 ml of 2.2 mg/ml acidic-extracted type I collagen solution (Corning) with pH buffered to 7.5 and loaded on a glass coverslip. After gelling for 30 min at 37°C, complete medium was added and collagen-embedded cells were incubated for 24 hrs at 37°C in 5% CO_2. After fixation in 4% paraformaldehyde in PBS at 37°C for 30 min, samples were incubated with ColI-$^{3/4}$C pAb (2.5 μg/ml) for 2 hrs at 4°C, washed extensively with PBS, and counterstained with Cy3-conjugated anti-rabbit IgG antibodies, 4',6-diamidino-2-phenylindole (DAPI), and Alexa Fluor-phalloidin to visualize cell shape. Image acquisition was performed with an A1R confocal microscope (Nikon) with a 40x oil objective. Quantification of the degradation spots was performed with a home-made plugin in ImageJ [9].

Multicellular spheroid outgrowth in 3D Matrigel

For the analysis of invasive outgrowth of MCF10DCIS.om cells, an overlay basement membrane assay was performed. Briefly, 6-well plates were coated with 12 mg/ml native Matrigel and allowed to solidify for 20 min at 37°C. MCF10DCIS.com cells (3 x 10^5 cells) were seeded as single cells onto the solidified basement membrane. After 7 days, cells were imaged in triplicate for development of invasive outgrowths by differential interference contrast (DIC) imaging using a 20x objective. Invasive growths were defined as consisting of two or more cells migrating away from their structure of origin. A minimum of 20 images were analyzed for each condition.

Analysis of MT1-MMP cell surface expression

For surface detection of MT1-MMP, MCF10DCIS.com cells treated with siRNAs against NME1, NME2, and non-targeting siRNA (scrambled) for 72 hrs, ablated for NME1 and NME2 by the CRISPR/Cas9 technology, and MDA-MB-435 cells stably

overexpressing NME1 and the empty vector were cultured, collected with 5 mM EDTA in PBS with 2% BSA and surface labeled with mouse mAb against MT1-MMP (clone 2D7) and AlexaFluor-488-conjugated anti-mouse IgG secondary antibodies. Specifically, cells were blocked with 2% BSA in PBS for 1h, incubated with the primary antibody, washed three times with PBS, incubated with the secondary antibody, and washed an additional three times with PBS. To ensure surface labeling, all solutions were ice-cold and the cells were kept on ice during all incubation steps. Following staining, cells were fixed in 2% paraformaldehyde and kept in the dark until analysis. Control cells were stained in parallel with secondary antibody only to reveal background. Analysis was done using the X Flow Cytometer. The mean fluorescence intensity for control cells (secondary antibody staining only) was subtracted from the mean fluorescence intensity for each cell line population and the results are graphed as percentage of expression relative to the non-targeting siRNA or the non-targeting CRISPR/Cas9 or to the empty vector.

In situ proximity ligation assay (PLA)

To monitor the subcellular localization of protein-protein interactions at single molecule resolution, an in situ proximity ligation assay (PLA) was performed as previously described [10]. Cells grown on coverslips were fixed with cold methanol and then incubated with primary antibodies. Secondary antibodies tagged with short DNA oligonucleotides were added. Hybridization, ligation, amplification and detection were realized according to the manufacturer's protocol (Sigma-Aldrich, St. Louis, MO). Briefly, secondary antibodies were incubated in pre-heated humidity chamber for 1 hr at 37°C. Ligation was performed with a ligase-containing ligation solution for 30 min at 37°C. Finally, amplification was performed with a polymerase-containing amplification

solution for 100 min at 37°C. PLA signal corresponds to the Cy3 fluorescence. Coverslips were analyzed on an inverted wide-field microscope.

Immunoprecipitation

Cells were lysed in 50 mM Tris-HCl pH 7.5, 137 mM NaCl, 10 mM MgCl2, 10% glycerol, 1% Triton-X100 with protease inhibitors and centrifuged at 16,000*g* for 10 min at 4°C. Supernatants were incubated with 2 μg of antibody for 2 hrs at 4°C and a 1:1 mixture of Protein-A and Protein-G Sepharose 4 Fast Flow (GE Healthcare) was added and further incubated for 1 hr at 4°C. Beads were washed three times in lysis buffer, and bound proteins were eluted in SDS sample buffer and analyzed by immunoblotting with the indicated antibodies.

Subcellular fractionation

Cells were scraped and resuspended in hypotonic buffer (10 mM HEPES, pH 7.5, 2.5 mM $MgCl_2$, 2 mM EGTA with a cocktail of protease inhibitors) by repeated passages through a 27G needle. The homogenate was centrifuged at 200*g* for 10 min at 4°C to yield a post-nuclear supernatant (PNS), which was centrifuged at 100,000*g* for 60 min at 4°C to yield supernatant and pellet fractions. Pellet was resuspended in hypotonic buffer to a volume equal to the supernatant volume, and equal volume of high-speed fractions and PNS were analyzed by immunoblotting using specific antibodies. The purified clathrin-coated vesicle (CCV) fraction was kindly provided by Dr E. Smythe (University of Sheffield, UK) [11].

Production and purification of recombinant proteins

The human MT1-MMP cytosolic tail (Cter) construct for bacterial expression was produced by Genscript. The synthetic DNA coding the MT1-MMP cytosolic tail (RRHGTPRRLLYCQRSLLDKV) was inserted into pGS-21a plasmid using NcoI cloning sites. The resulting pGS-21a-MT1-MMP-Cter plasmid allowed production of MT1-MMP-Cter peptide with a cleavable by enterokinase N-terminal His-GST tag. The recombinant His-GST-MT1-MMP-Cter protein expression was performed in Escherichia coli BL21(DE3) cells. Bacterial cells were grown at 37 °C in 2xYT medium, induced at an A600 nm of OD 0.6 by the addition of 0.5 mM isopropyl-1-thio-β-D-galactopyranoside, and harvested after 18 hrs at 20°C. The cell pellet was resuspended in 50 mM Tris, pH 8.0, 150 mM NaCl, 40 mM imidazole, 1 mM TCEP and protease inhibitor mix (CLAP/cOmplete from Sigma at 1 µg/ml), lysed by sonication, and centrifuged at 35000 x *g* for 1 h. The supernatant was loaded onto a HisTrap column (GE healthcare). After washing with 20 column volumes of the wash buffer (50 mM Tris, pH 8.0, 150 mM NaCl, 40 mM imidazole, 1 mM TCEP), the protein was eluted with 250 mM imidazole. The eluted fractions with the highest protein concentration were analyzed on SDS-PAGE, the fractions containing pure His-GST-MT1-MMP-Cter were pulled and diluted 5 times in the imidazole-free buffer resulting in the 10 mg/ml protein solution that was aliquoted, frozen in liquid nitrogen and stored at -80ºC. The purified His-GST-MT1-MMP-Cter integrity was confirmed by MALDI-TOF MS analysis. Recombinant NME1 was produced as described elsewhere [12]. Purified His-GST-MT1-MMP-Cter and NME1 proteins homogeneity and oligomerization states were controlled and confirmed using size exclusion chromatography (Superdex 200) combined with multi angle light scattering (Wyatt Technology). The recombinant NME1 formed the expected hexamers.

Pull-down assay

Purified GST or GST-MT1-MMP-Cter recombinant proteins (25 μg) were immobilized on Glutathione Sepharose 4B beads (GE Healthcare) (1:1 suspension) for 1 hr at 4°C on a rotating wheel. Beads were washed three times with pulldown buffer (25 mM Tris-HCl pH 7.4, 150 mM NaCl, 5 mM $MgCl_2$, 10% (v/v) glycerol, 1% (v/v) Nonidet P-40) and subsequently incubated with purified recombinant NME1 protein (50 μg) for 2 hrs at 4°C on a rotating wheel. Beads were washed four times with pulldown buffer and bound proteins were eluted with 1x Laemmli buffer and denatured at 95°C for 30 min, and then analyzed by immunoblotting.

MT1-MMP internalization

Cells were incubated for 1 hr on ice in the presence of 0.5 mg/ml NHS-SS-biotin (Pierce Chemical, Rockford, IL). Labeled cells were then washed and incubated at 37°C for 1 hr to allow for internalization of surface proteins. Samples were then washed and treated successively (20 min at 4°C) with a reducing solution (42 mM glutathione, 75 mM NaCl, 1 mM EDTA, 1% bovine serum albumin, and 75 mM NaOH) to strip biotinylated proteins from the cell surface. After a final wash, cells were lysed in 10 mM Tris, pH 7.2, 150 mM NaCl, 1 mM $CaCl_2$, 1 mM $MgCl_2$, and 1 % Nonidet P-40. Biotinylated proteins were captured with streptavidin-Sepharose beads (Pierce Chemical), and recovered complexes were resolved under reducing conditions by SDS-PAGE followed by Western blot analysis. The total pool of biotinylated MT1-MMP was determined in samples where the incubation step with the reducing solution was omitted [13].

Statistical analysis

Analyses were performed using R software, 3.5.0 version (http://cran.rproject.org). Boxplots were generated with the R ggplot2 package. All statistical tests were two-sided. *P* values of 0.05 or below were considered significant. Comparison of H-scores between matched in situ and invasive or microinvasive lesions was performed using paired Student's t-test. Comparison of H-scores between two different breast molecular subtypes was performed using Welch two samples t-test (Welch's t-test is an adaptation of the Student's t-test and is more reliable when the two independent samples have unequal sample sizes). Membranous MT1-MMP and total NME1 H-scores were compared between groups by ANOVA test. Comparison of in situ or invasive tumors in glands injected with "NT", "KO NME1(#A)", "KO NME1(#B)", "KO NME2(#A)" or "KO NME2(#B)" clones was performed using the Chi2 test. The distributions of tumor area (mm^2) in mammary glands in mice injected with "NT", "KO NME1(#A)", "KO NME1(#B)", "KO NME2(#A)" or "KO NME2(#B)" clones were compared using a non-parametric method by ranks (Kruskall-Wallis test) because of small sample size. Comparisons were also performed according to the tumor type (in situ or invasive tumor, respectively). For all other comparisons, the unpaired Student's t-test was used.

Supplemental references

1 Hotary K, Allen E, Punturieri A, Yana I, Weiss SJ. Regulation of cell invasion and morphogenesis in a three-dimensional type I collagen matrix by membrane-type matrix metalloproteinases 1, 2, and 3. *J Cell Biol* 2000; 149: 1309-1323.

2 Boissan M, Wendum D, Arnaud-Dabernat S, Munier A, Debray M, Lascu I *et al*. Increased lung metastasis in transgenic NM23-Null/SV40 mice with hepatocellular carcinoma. *J Natl Cancer Inst* 2005; 97: 836-845.

3 Chenard MP, Lutz Y, Mechine-Neuville A, Stoll I, Bellocq JP, Rio MC *et al*. Presence of high levels of MT1-MMP protein in fibroblastic cells of human invasive carcinomas. *Int J Cancer* 1999; 82: 208-212.

4 Lodillinsky C, Infante E, Guichard A, Chaligne R, Fuhrmann L, Cyrta J *et al*. p63/MT1-MMP axis is required for in situ to invasive transition in basal-like breast cancer. *Oncogene* 2016; 35: 344-357.

5 Prat A, Cheang MC, Martin M, Parker JS, Carrasco E, Caballero R *et al*. Prognostic significance of progesterone receptor-positive tumor cells within immunohistochemically defined luminal A breast cancer. *J Clin Oncol* 2013; 31: 203-209.

6 Wolff AC, Hammond ME, Schwartz JN, Hagerty KL, Allred DC, Cote RJ *et al*. American Society of Clinical Oncology/College of American Pathologists

guideline recommendations for human epidermal growth factor receptor 2 testing in breast cancer. *Arch Pathol Lab Med* 2007; 131: 18-43.

7 Behbod F, Kittrell FS, LaMarca H, Edwards D, Kerbawy S, Heestand JC *et al*. An intraductal human-in-mouse transplantation model mimics the subtypes of ductal carcinoma in situ. *Breast Cancer Res* 2009; 11: R66.

8 De Wever O, Hendrix A, De Boeck A, Westbroek W, Braems G, Emami S *et al*. Modeling and quantification of cancer cell invasion through collagen type I matrices. *Int J Dev Biol* 2010; 54: 887-896.

9 Monteiro P, Rosse C, Castro-Castro A, Irondelle M, Lagoutte E, Paul-Gilloteaux P *et al*. Endosomal WASH and exocyst complexes control exocytosis of MT1-MMP at invadopodia. *J Cell Biol* 2013; 203: 1063-1079.

10 Soderberg O, Gullberg M, Jarvius M, Ridderstrale K, Leuchowius KJ, Jarvius J *et al*. Direct observation of individual endogenous protein complexes in situ by proximity ligation. *Nat Methods* 2006; 3: 995-1000.

11 Semerdjieva S, Shortt B, Maxwell E, Singh S, Fonarev P, Hansen J *et al*. Coordinated regulation of AP2 uncoating from clathrin-coated vesicles by rab5 and hRME-6. *J Cell Biol* 2008; 183: 499-511.

12 Mocan I, Georgescauld F, Gonin P, Thoraval D, Cervoni L, Giartosio A *et al*. Protein phosphorylation corrects the folding defect of the neuroblastoma

(S120G) mutant of human nucleoside diphosphate kinase A/Nm23-H1. *Biochem J* 2007; 403: 149-156.

13 Li XY, Ota I, Yana I, Sabeh F, Weiss SJ. Molecular dissection of the structural machinery underlying the tissue-invasive activity of membrane type-1 matrix metalloproteinase. *Mol Biol Cell* 2008; 19: 3221-3233.

Supplemental Figure legends

Supplemental Figure S1. Specificity validation of NME1 and -NME2 antibodies

(A) Left panel: NME1 expression was analyzed by immunoblotting using home-made NME1 pAb in MDA-MB-435 and MDA-MB-231 cells stably overexpressing NME1 (NME1) or in cells transfected with a control vector (CTRL), and in MCF10DCIS.com cells treated with a NME1-targeting siRNA (siNME1) or scramble siRNA (Scr). Right panel: the same samples were analyzed with NME1 mAb from OriGene. (**B**) Enzyme-linked immunosorbent assay (ELISA) using purified recombinant human $NME1_r$ or $NME2_r$ proteins and 10^{-3}, 10^{-4} and 10^{-5} dilutions of NME2 mAb (Kamiya Biomedical Company). **(C)** Western blotting analysis using purified recombinant human $NME1_r$, $NME2_r$, $NME3_r$ or $NME4_r$ proteins revealed with NME2 mAb as in B. **(D)** Western blotting analysis of HeLa cell lysates silenced for NME2 or scramble siRNA-treated (Scr) with NME2 mAb as in B. Alpha-tubulin was used as a loading control. Molecular weights are in kDa.

Supplemental Figure S2. NME1 expression in synchronous in situ and invasive components of breast tumors

(A-B) Representative IHC staining of NME1 in breast peritumoral tissues and in synchronous in situ and invasive components from two breast carcinoma biopsies using home-made NME1 pAb. Arrowheads point to plasma membrane NME1 staining. Scale bar, 50 μm.

Supplemental Figure S3. NME1 is downregulated in invasive relative to synchronous in situ components irrespective of the breast cancer subgroup

(A-C) Comparison of NME1 levels using the H-score method in in situ and invasive components of the breast cancer cohort (N=156 breast tumor clinical specimens) segregated in the different Luminal A & B, HER2+ and TNBC subgroups. The median of each H-score distribution is represented (red bar). **(D, E)** Comparison of NME1 levels in the in situ (D) or invasive components (E) of Luminal A & B and TNBC breast carcinomas. *** $P < 0.001$; * $P < 0.05$. The median of each H-score distribution is represented (red bar).

Supplemental Figure S4. Confirmation of biphasic up- and down-regulation of NME1 expression using NME1 mAb from OriGene

(A-B) Representative IHC staining of NME1 in breast peritumoral tissues and in synchronous in situ and invasive components from two breast carcinoma biopsies using Origene NME1 mAb. Scale bar, 25 μm.

Supplemental Figure S5. NME1 expression is downregulated in microinvasive breast carcinomas

(A-B) Two representative examples of NME1 IHC staining of synchronous in situ and microinvasive breast carcinomas. Arrowheads point to plasma membrane staining. Scale bar, 50 μm. **(C-E)** Comparison of total (C), cytoplasmic (D) and plasma membrane (E) NME1 levels (H-score) in synchronous in situ and microinvasive breast carcinomas. *** $P < 0.001$; ** $P < 0.01$. The median of each H-score distribution is represented (red bar).

Supplemental Figure S6. Up-regulation of NME2 in breast cancer

(A-B) Representative NME2 IHC staining in breast peritumoral tissues and synchronous in situ and invasive components from two breast carcinoma biopsies. Arrows point to NME2 apical staining in peritumoral epithelial tissue. Arrowheads point to plasma membrane staining in breast carcinoma cells. Scale bar, 25 μm. (**C, E and G**) Comparison of total (C), cytoplasmic (E) and plasma membrane (G) NME2 levels using the H-score method in in situ breast carcinomas as compared to adjacent peritumoral tissues. *** $P < 0.001$; ns, not significant. **(D, F and H)** NME2 levels were compared in synchronous in situ and invasive components of breast tumor biopsies. ns, not significant. The median of each H-score distribution is represented (red bar).

Supplemental Figure S7. Additional examples of anti-correlated NME1 and MT1-MMP staining in human breast tumors

(A, B) Representative immunostaining of serial sections of synchronous in situ (A) and invasive (B) components of human breast carcinoma (case #2) using NME1 pAb and MT1-MMP mAb. Tumor foci are delineated by a dashed line. Scale bar, 25 μm.

Supplemental Figure S8. Definition of cut-off values for membranous MT1-MMP and total NME1 H-score variables in the overall breast cancer cohort

(A-D) Cut-off values for membranous MT1-MMP and total NME1 H-score variables were calculated using normal mixture modeling in in situ (A, B) and invasive (C, D) components of the overall human breast tumor cohort. The same cut-off values were used for in situ and invasive components. Threshold membranous MT1-MMP: 125; threshold total NME1: 300.

Supplemental Figure S9. Validation of NME1 and NME2 knockout in MCF10DCIS.com clones

Fraction of sequencing reads aligned to the reference amplicon sequence and for which an insertion or deletion was observed. For both NME1 and NME2 genes, two sites (referred to as A and B) were targeted for edition by CRISPR/Cas9 in MCF10DCIS.com cells. Corresponding targeted sites were sequenced after amplification with appropriate primers (see Table S1). Top labels refer to the different MCF10DCIS.com cell populations. Bar colors and x axis labels refer to the amplicon. Parental, MCF10DCIS.com cells not subjected to CRISPR/Cas9 edition; NT, MCF10DCIS.com cells not targeted, i.e. cells subjected to CRISPR/Cas9 edition with no guide; KO NME1(#A) and (#B), MCF10DCIS.com cells subjected to CRISPR/Cas9 edition of NME1 gene for which two sites A and B were targeted, respectively; KO NME2(#A) and (#B), MCF10DCIS.com cells subjected to CRISPR/Cas9 edition of NME2 gene for which two sites A and B were targeted, respectively.

Supplemental Figure S10. Increased proliferation rate in NME2 knockout intraductal tumor xenografts

(A) Cell proliferation marker, PCNA, (green) and DAPI (blue) immunofluorescence staining of intraductal tumor xenograft tissue sections of control (NT) and knockout MCF10DCIS.com clones. Analysis was performed 4 weeks after nipple injection. Scale bar, 50 µm. **B)** The percentage of PCNA-positive nuclei was determined from three different fields from three independent tumors. Error bars are the standard error of the mean (SEM). *** $P < 0.001$.

Supplemental Figure S11. Validation of MT1-MMP surface labeling by FACS

(A, C) Lysates of MCF10DCIS.com cells knockdown for MT1-MMP upon shRNA expression (A) or overexpressing MT1-MMPmCherry (C) were analyzed by immunoblotting with MT1-MMP mAb. Mock-treated (A) or parental (C) MCF10DCIS.com cell lysates were used as control. Molecular weights are in kDa. **(B, D)** Representative FACS profiles generated by surface MT1-MMP labeling in cells silenced (A) or overexpressing (C) MT1-MMP as compared to control cells (continuous line).

Supplemental Figure S12. Immunoblotting analysis of MT1-MMP expression in knockdown cells

(A) Lysates of control non-KO MCF10DCIS.com cells (NT) or of two independent clones knockout for NME1 or NME2 treated (+) or not (-) with MT1-MMP siRNA were analyzed by immunoblotting with the anti-MT1-MMP antibodies. Alpha-tubulin was used as a loading control. **(B)** Lysates of MCF10DCIS.com cells treated with control scrambled siRNA (Scr), or siRNA specific for NME1 or MT1-MMP were analyzed by immunoblotting with the indicated antibodies. GAPDH was used as a loading control. Molecular weights are in kDa.

Supplemental Figure S13. Cell fractionation and co-immunoprecipitation analysis of NME2

(A) After homogenization, a post-nuclear supernatant (PNS) of MCF10DCIS.com cells was ultracentrifuged to produce soluble (Supernatant) and membrane (Pellet) fractions. Proteins corresponding to equivalent cell-number were loaded in each lane and analyzed by immunoblotting with the indicated antibodies. The transferrin receptor (TfR) and the α-adaptin subunit of the AP-2 clathrin adaptor complex were recovered

in the membrane pellet fraction. NME2 partitioned both in the cytosolic and membrane fractions similar to NME1 (see Figure 4A). **(B)** PNS and clathrin-coated vesicle (CCV) fractions (10 μg) isolated from porcine brain were analyzed by immunoblotting with the indicated antibodies. **(C)** Lysates of control MCF10DCIS.com cells or cells knocked out for NME2 (clone #A and #B) were immunoprecipitated with NME2 antibodies or control IgGs followed by immunoblotting analysis with MT1-MMP antibodies. 1% of total lysate was loaded as a control (input). Molecular weight markers are indicated (in kDa).

Primer name	Adapter sequence	Locus specific primer sequence
NME1A-Fwd	TCGTCGGCAGCGTCAGATGTGTATAAGAGACAG	AATAGTTGCCAGATTTTCTGCTGT
NME1A-Rev	GTCTCGTGGGCTCGGAGATGTGTATAAGAGACAG	GGGAAAAATACCAAAATCTCACCT
NME1B-Fwd	TCGTCGGCAGCGTCAGATGTGTATAAGAGACAG	CAGTGTGGAGAATGAATTGGGTTA
NME1B-Rev	GTCTCGTGGGCTCGGAGATGTGTATAAGAGACAG	AGTATCCCACACAGGCACACTC
NME2A-Fwd	TCGTCGGCAGCGTCAGATGTGTATAAGAGACAG	GCGTGGTGGGGGAGGAG
NME2A-Rev	GTCTCGTGGGCTCGGAGATGTGTATAAGAGACAG	GGAGACGGGGGCGAGTTACC
NME2B-Fwd	TCGTCGGCAGCGTCAGATGTGTATAAGAGACAG	GACTTGCTAATGGGAGGTTCAGAG
NME2B-Rev	GTCTCGTGGGCTCGGAGATGTGTATAAGAGACAG	CAAAGAACACTGAGCACTTTTTCC

Table S1: Nextera XT adapter overhangs sequences and locus specific primer sequences used for the edition of NME1 and NME2 genes by CRISPR/Cas9.
For both NME1 and NME2 genes, two sites (referred to as A and B) were targeted for edition by CRISPR/Cas9 in MCF10DCIS.com cells. Corresponding targeted sites were sequenced after amplification with appropriate primers.

FEATURES	Invasive carcinoma<br><br>(n=156)	Microinvasive carcinoma<br>(n=37)
Age (years)		
≤ 50	72 (46,2%)	9 (24,3%)
> 50	84 (53,9%)	28 (75,7%)
Menopausal status		
Premenopausal	63 (40,4%)	9 (24,3%)
Postmenopausal	81 (51,9%)	26 (70,3%)
Unknown	12 (7,7%)	2 (8,1%)
Histological grade (invasive tumors)		
I	28 (18%)	x
II	60 (38,5%)	x
III	66 (42,3%)	x
Unknown	2 (1,3%)	x
Nuclear grade (CCIS & Mic)		
High	x	30 (81,1%)
Non high	x	7 (18,9%)
Histological subtype		
Ductal carcinoma	152 (97,5%)	x
Others	4 (2,5%)	x
Tumour size (cm)		
Tis	x	x
T1mic	x	37 (100%)
T1 (<2)	114 (73,1%)	x
T2 (2 - 5)	36 (23,1%)	x
T3 (>5)	6 (3,9%)	x
N stage		
N0	79 (50,6%)	x
N1	51 (32,7%)	x
N2	20 (12,8%)	x
N3	4 (2,6%)	x
Unknown	2 (1,3%)	x
ER		
Positive	93 (59,6%)	18 (48,6%)
Negative	63 (40,4%)	19 (51,4%)
PR		
Positive	81 (51,9%)	14 (37,9%)
Negative	74 (47,4%)	23 (62,1%)
Unknown	1 (0,7%)	x
HER2		
Positive	43 (27,6%)	15 (40,6%)
Negative	113 (72,5%)	22 (59,4%)
Ki67		
Positive (>20%)	111 (71,2%)	25 (67,6%)
Negative (<20%)	44 (28,2%)	12 (32,4%)
Unknown	1 (0,7%)	x
Molecular subtype		
TNBC	23 (14,8%)	3 (8,1%)

HER2	39 (25%)	14 (37,8%)
Luminal A	49 (31,4%)	8 (21,6%)
Luminal B	41 (26,3%)	8 (21,6%)
Luminal B / HER2	4 (2,6%)	4 (10,8%)

Table S2: Clinicopathological parameters of 156 invasive breast tumors and 37 microinvasive breast tumors

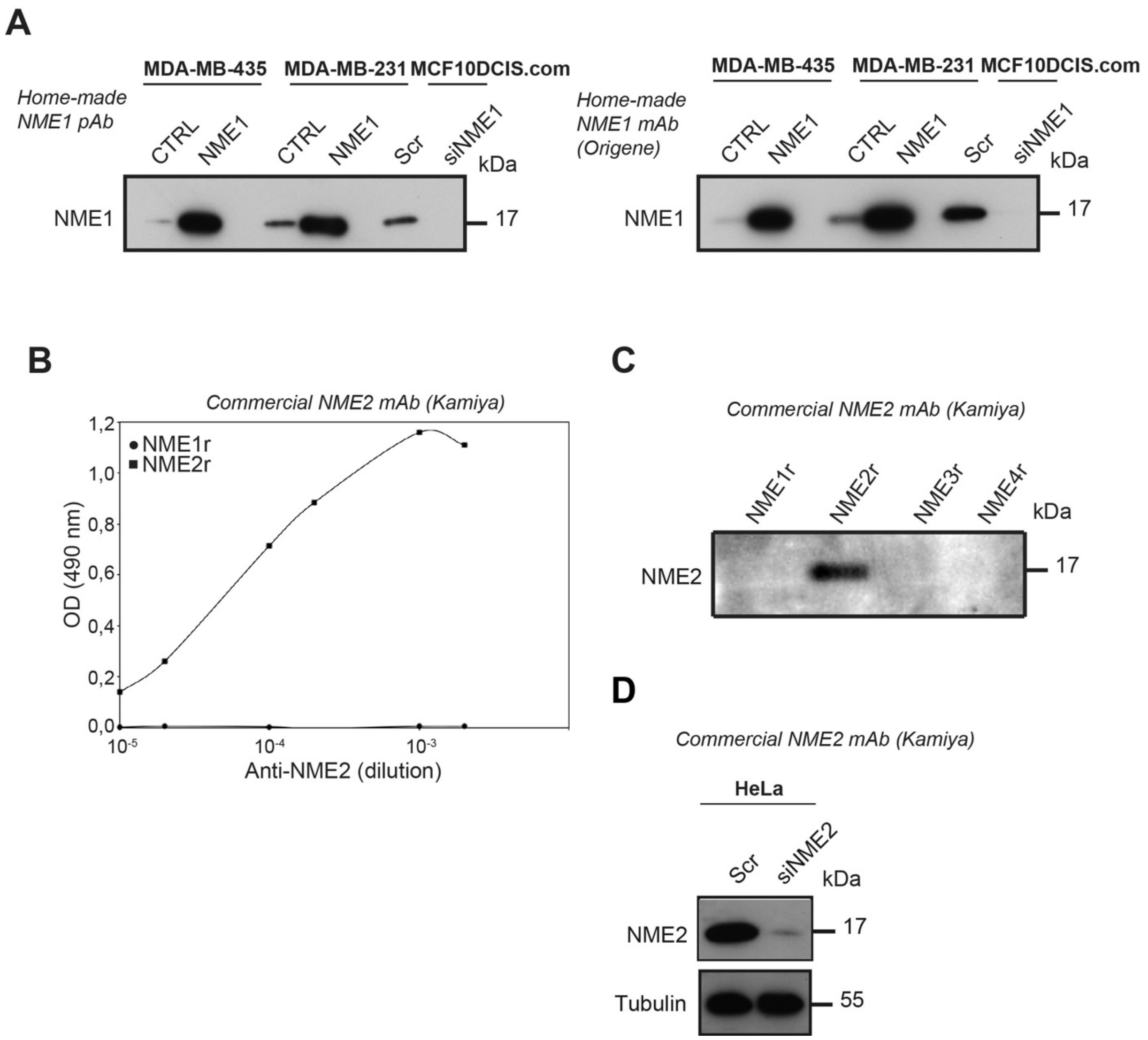
A
MDA-MB-435
MDA-MB-231
MCF10DCIS.com
Home-made NME1 pAb
CTRL
NME1
CTRL
NME1
Scr
siNME1
kDa
NME1
17
Home-made NME1 mAb (Origene)
B
Commercial NME2 mAb (Kamiya)
NME1r
NME2r
OD (490 nm)
Anti-NME2 (dilution)
C
Commercial NME2 mAb (Kamiya)
NME1r
NME2r
NME3r
NME4r
NME2
D
Commercial NME2 mAb (Kamiya)
HeLa
Scr
siNME2
Tubulin
55

Supplemental Figure 2

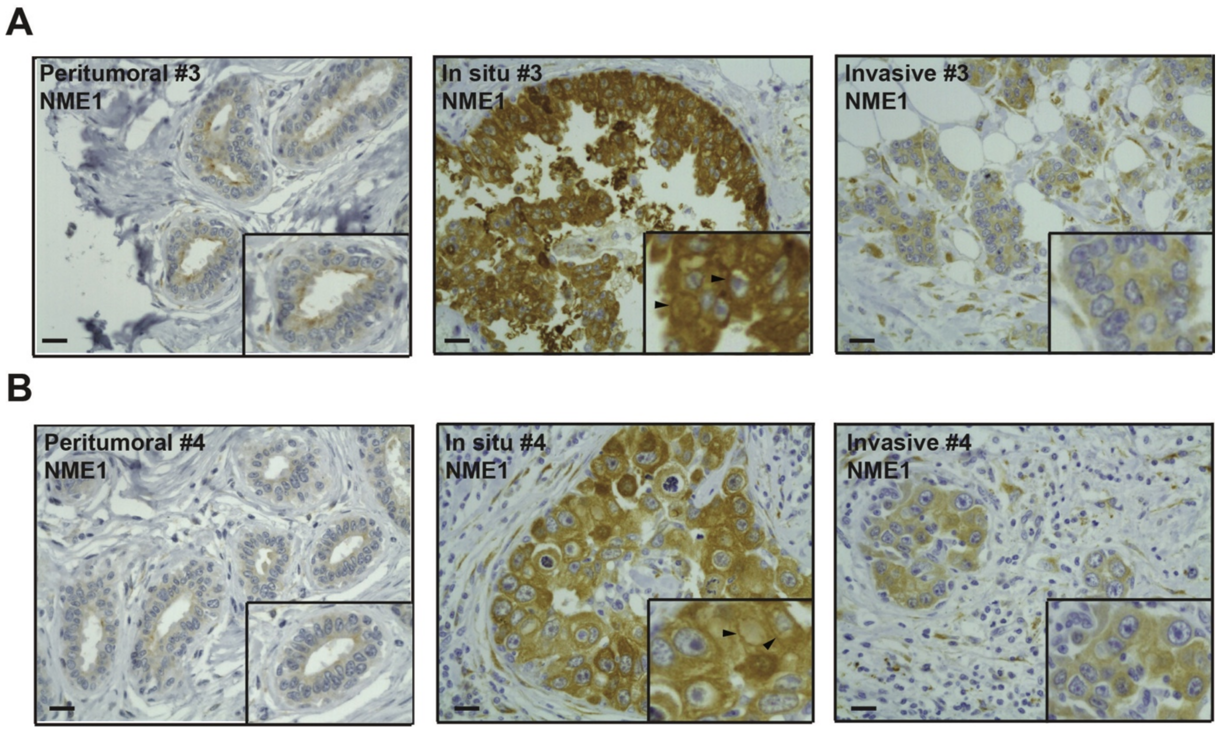

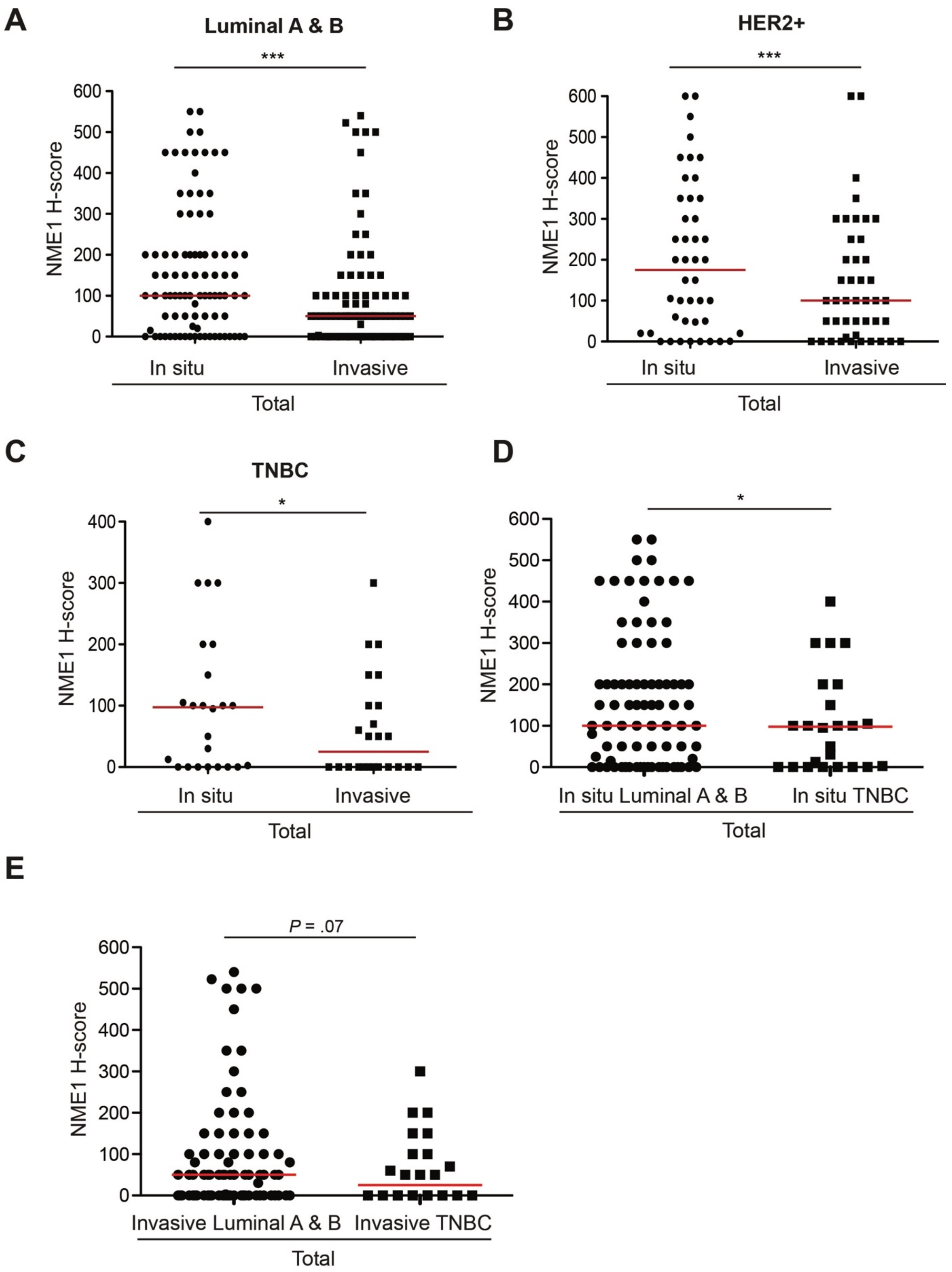
A
Luminal A & B
***
NME1 H-score
600
500
400
300
200
100
0
In situ
Invasive
Total
B
HER2+
***
NME1 H-score
In situ
Invasive
Total
C
TNBC
*
NME1 H-score
400
300
200
100
0
In situ
Invasive
Total
D
*
NME1 H-score
In situ Luminal A & B
In situ TNBC
Total
E
P = .07
NME1 H-score
Invasive Luminal A & B
Invasive TNBC
Total

Supplemental Figure 4

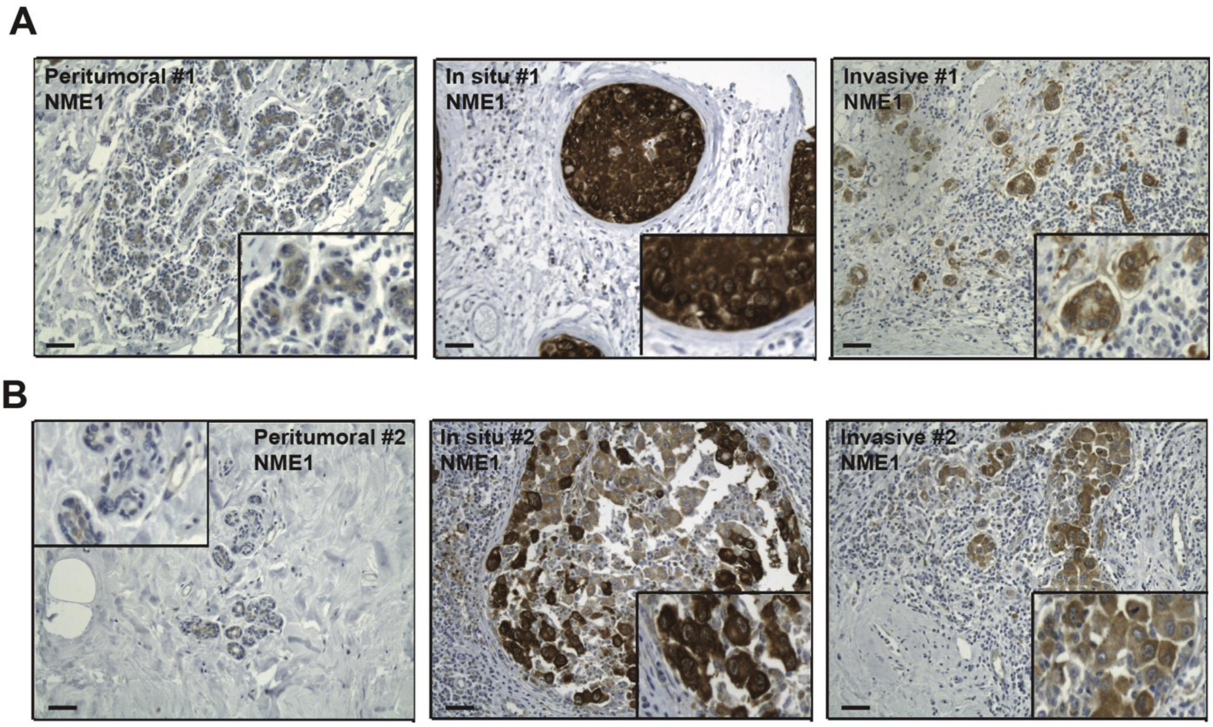

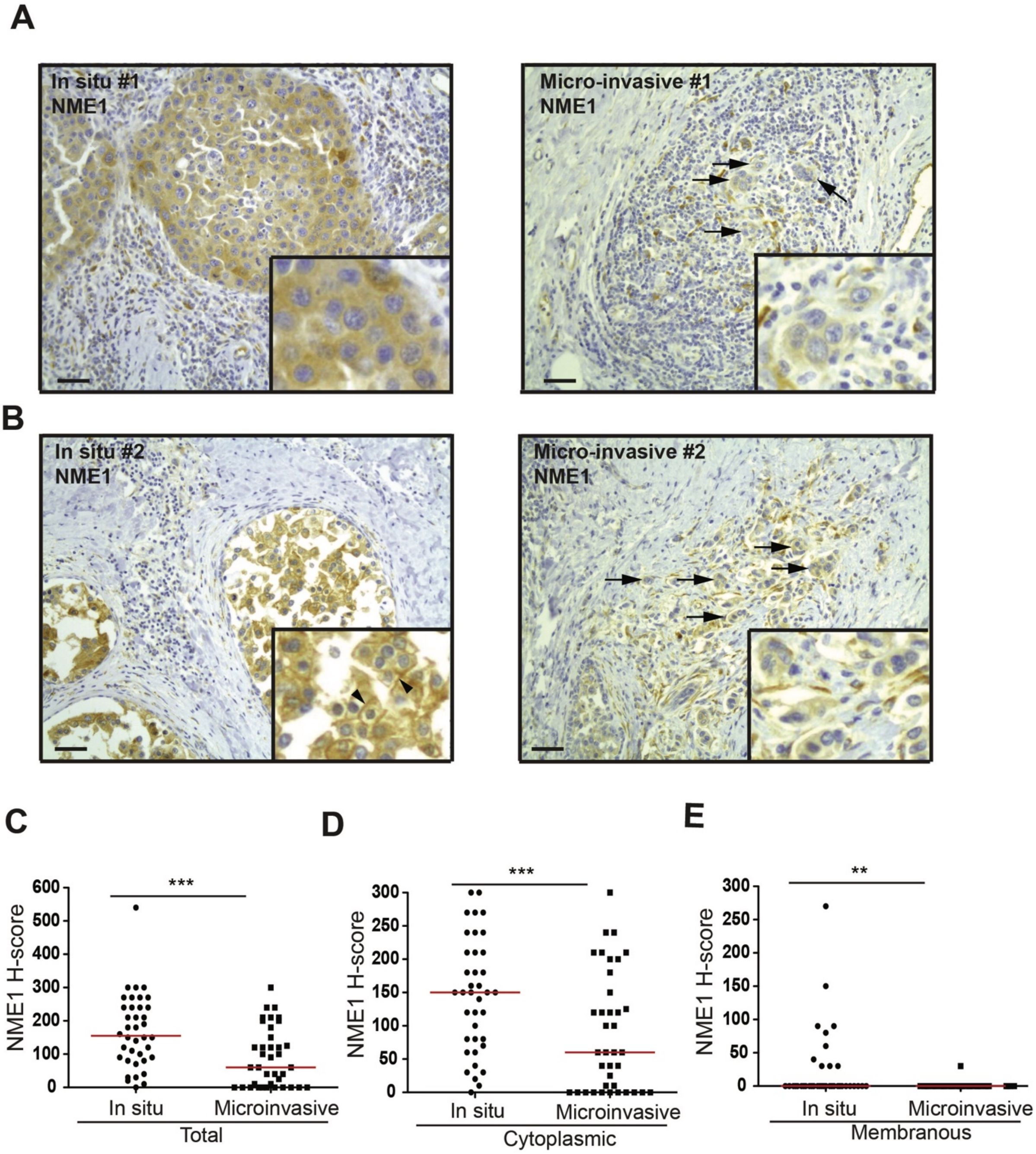
A
In situ #1
NME1
Micro-invasive #1
NME1
B
In situ #2
NME1
Micro-invasive #2
NME1
C
NME1 H-score
***
In situ
Microinvasive
Total
D
NME1 H-score
***
In situ
Microinvasive
Cytoplasmic
E
NME1 H-score
**
In situ
Microinvasive
Membranous

Supplemental Figure 6

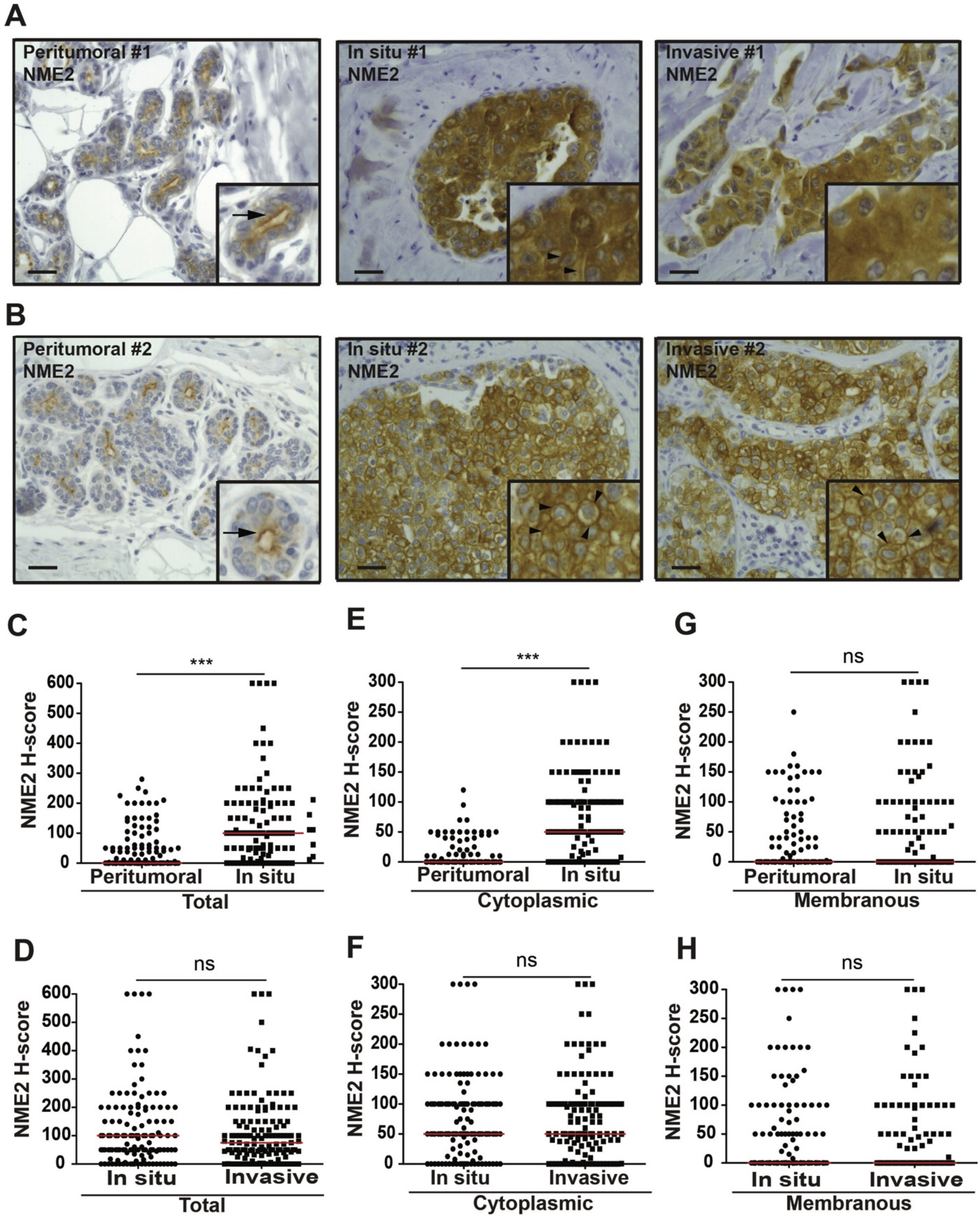

Supplemental Figure 7

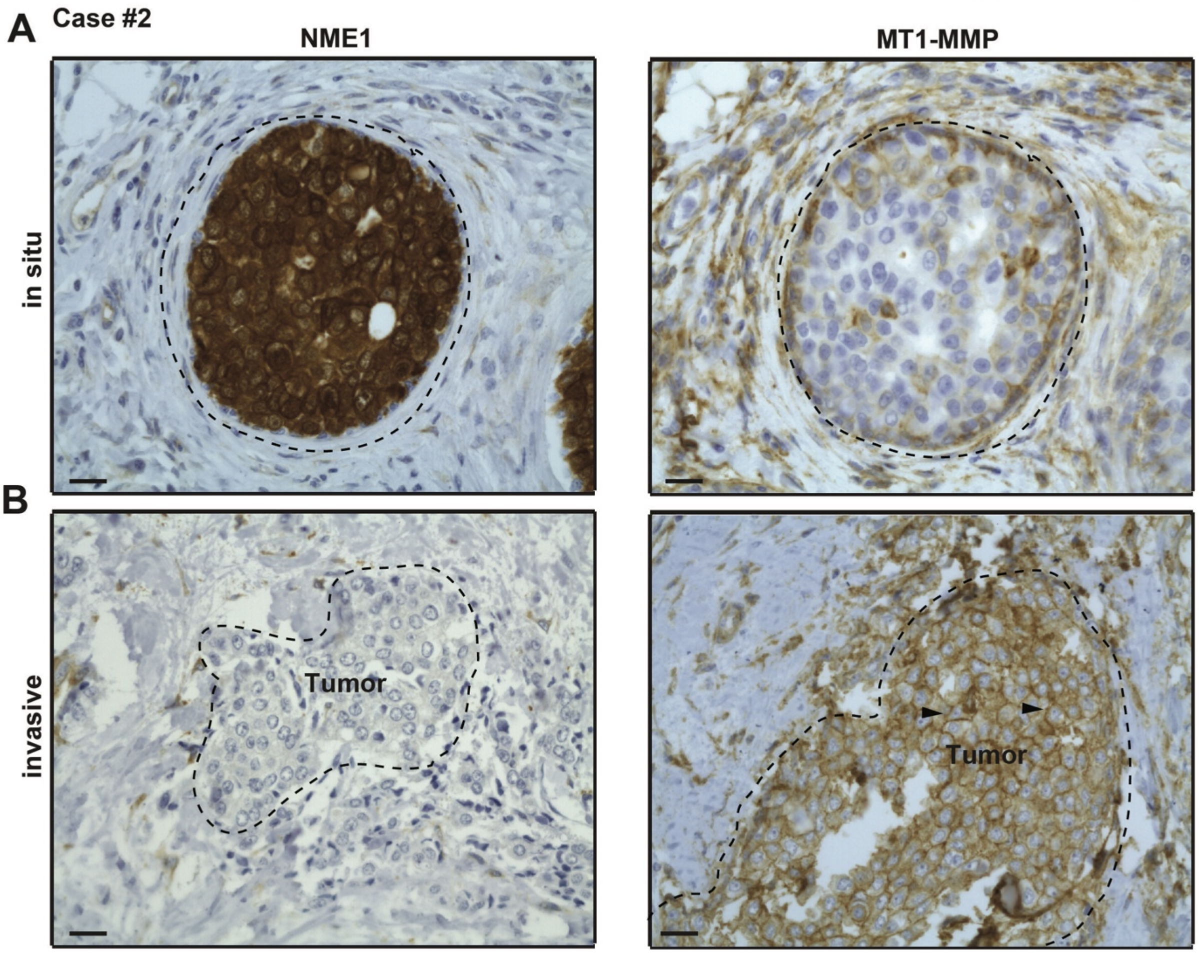

Supplemental Figure 8

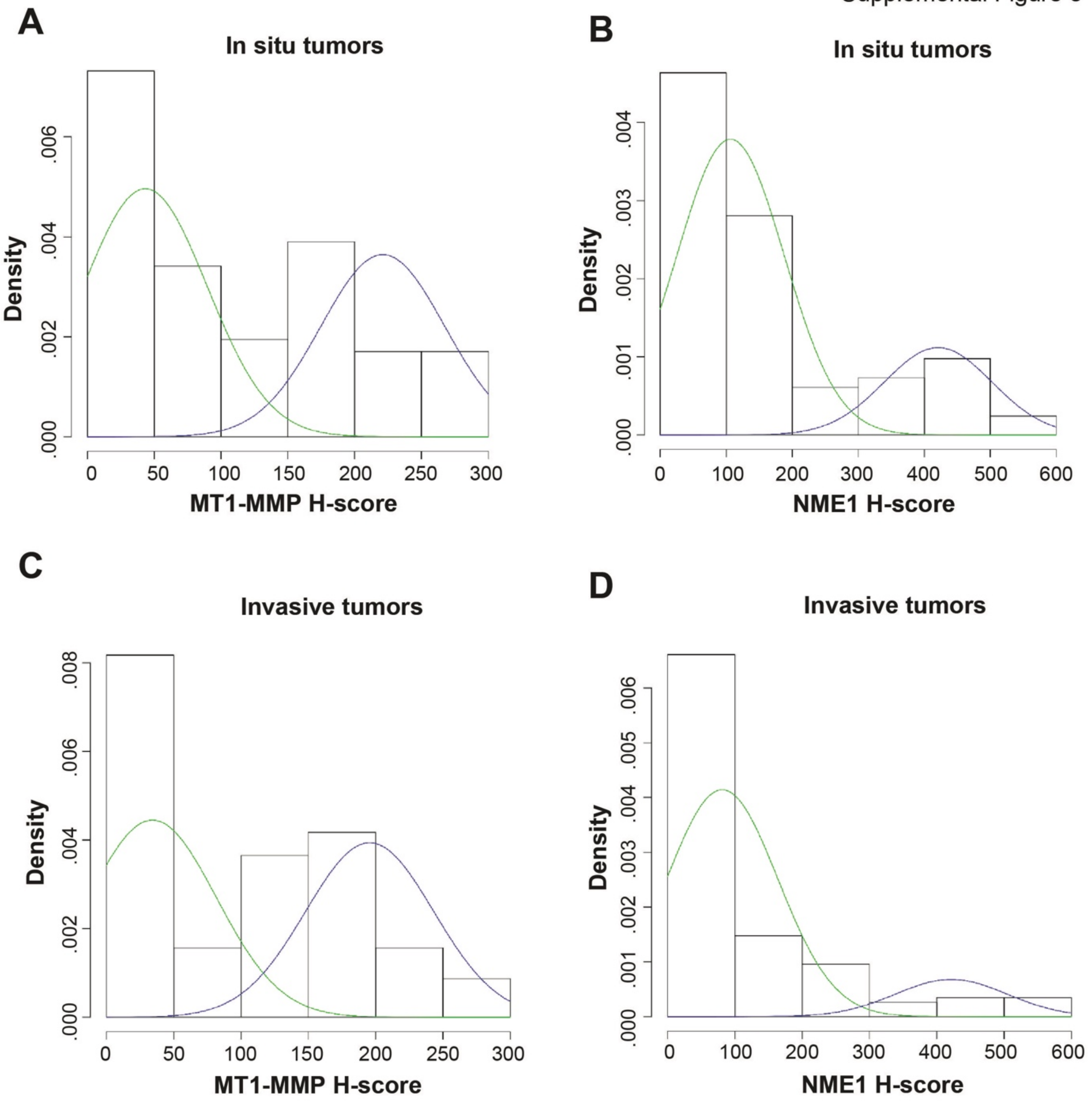

Supplemental Figure 9

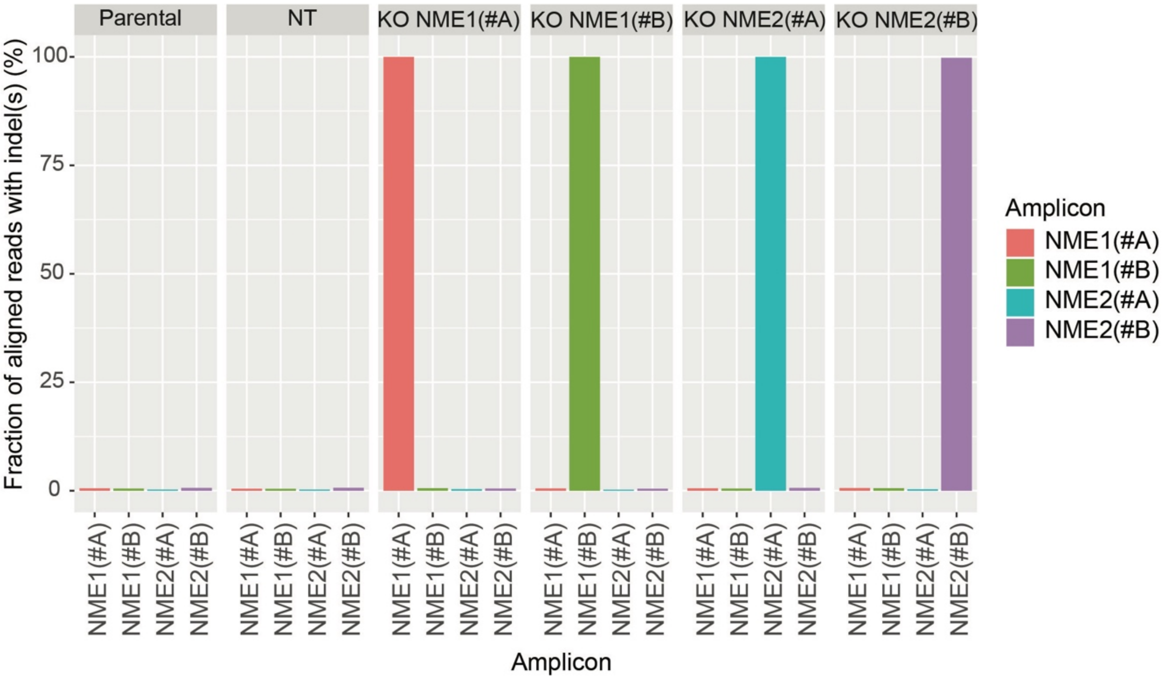

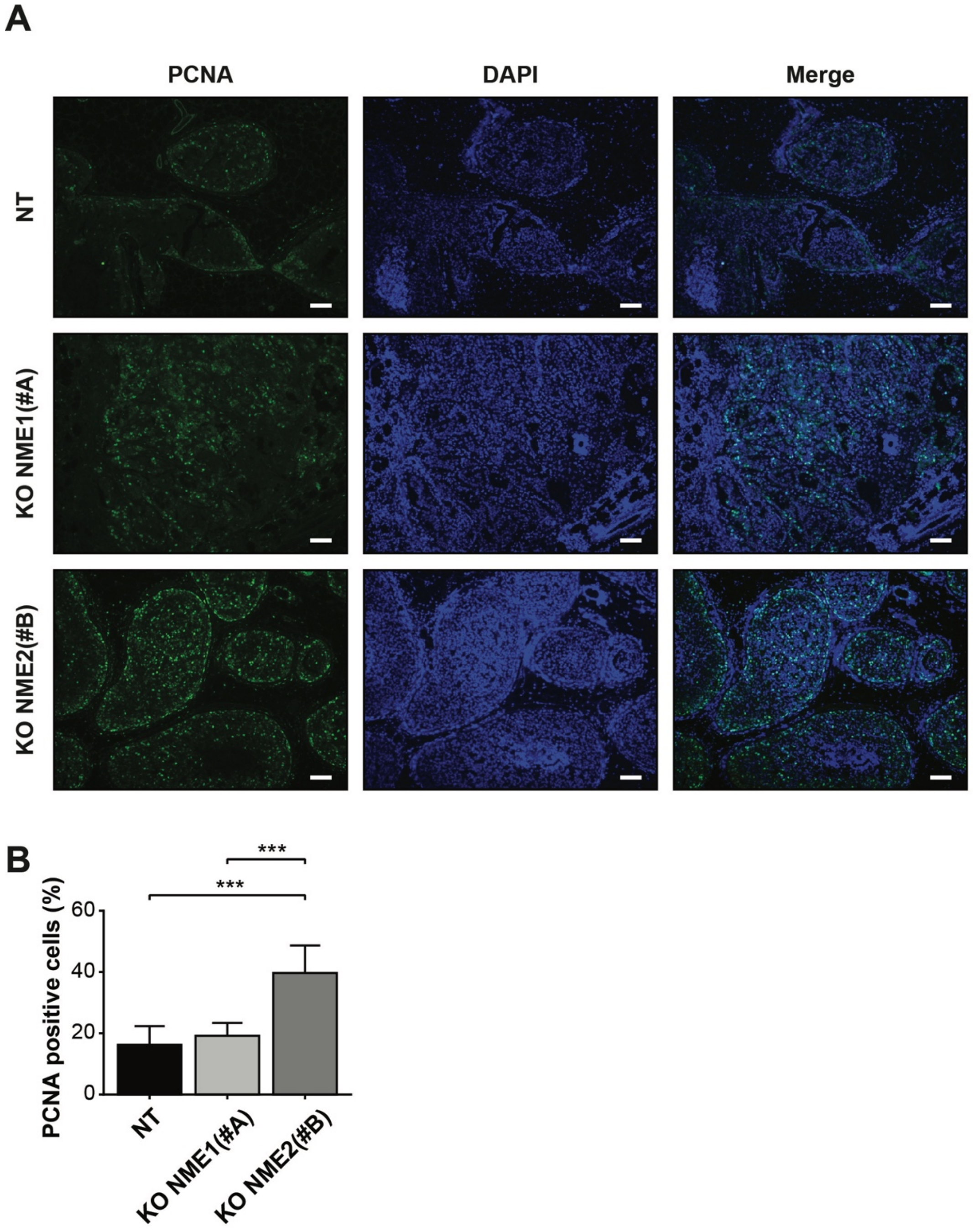
A
PCNA
DAPI
Merge
NT
KO NME1(#A)
KO NME2(#B)
B
***
***
PCNA positive cells (%)
60
40
20
0
NT
KO NME1(#A)
KO NME2(#B)

Supplemental Figure 11

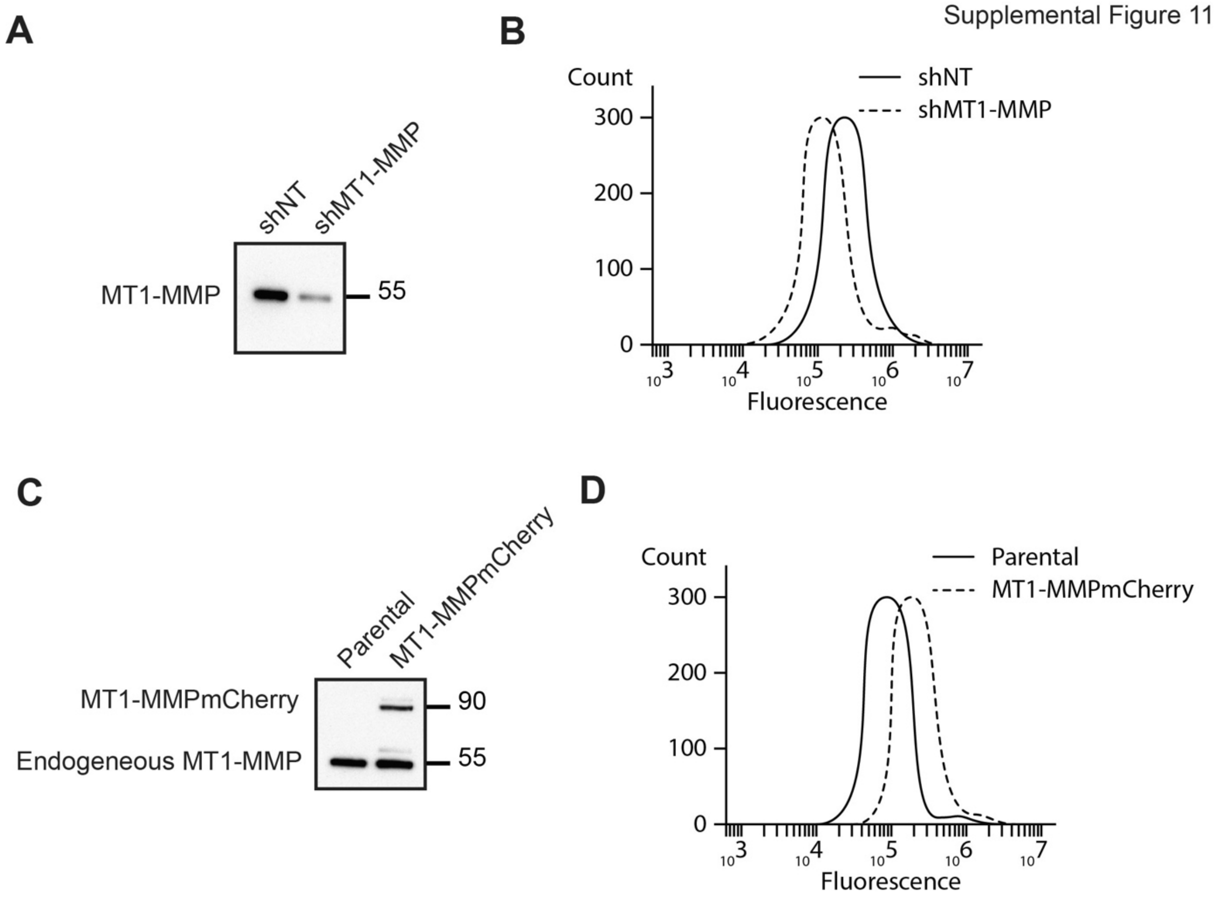

Supplemental Figure 12

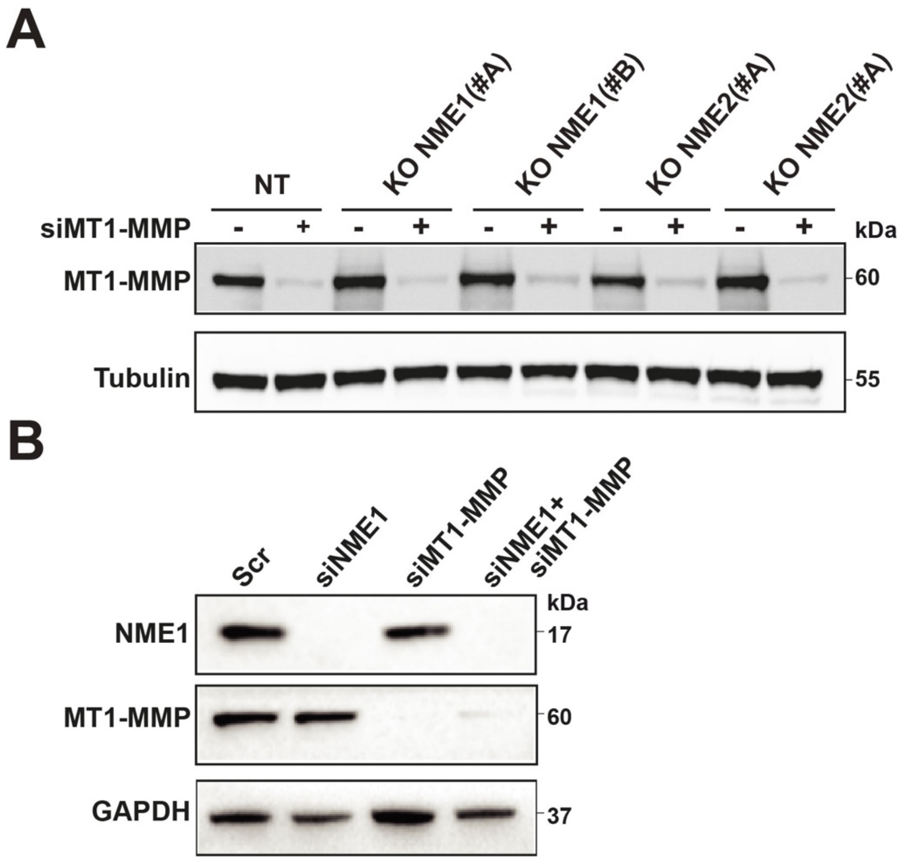

Supplemental Figure 13

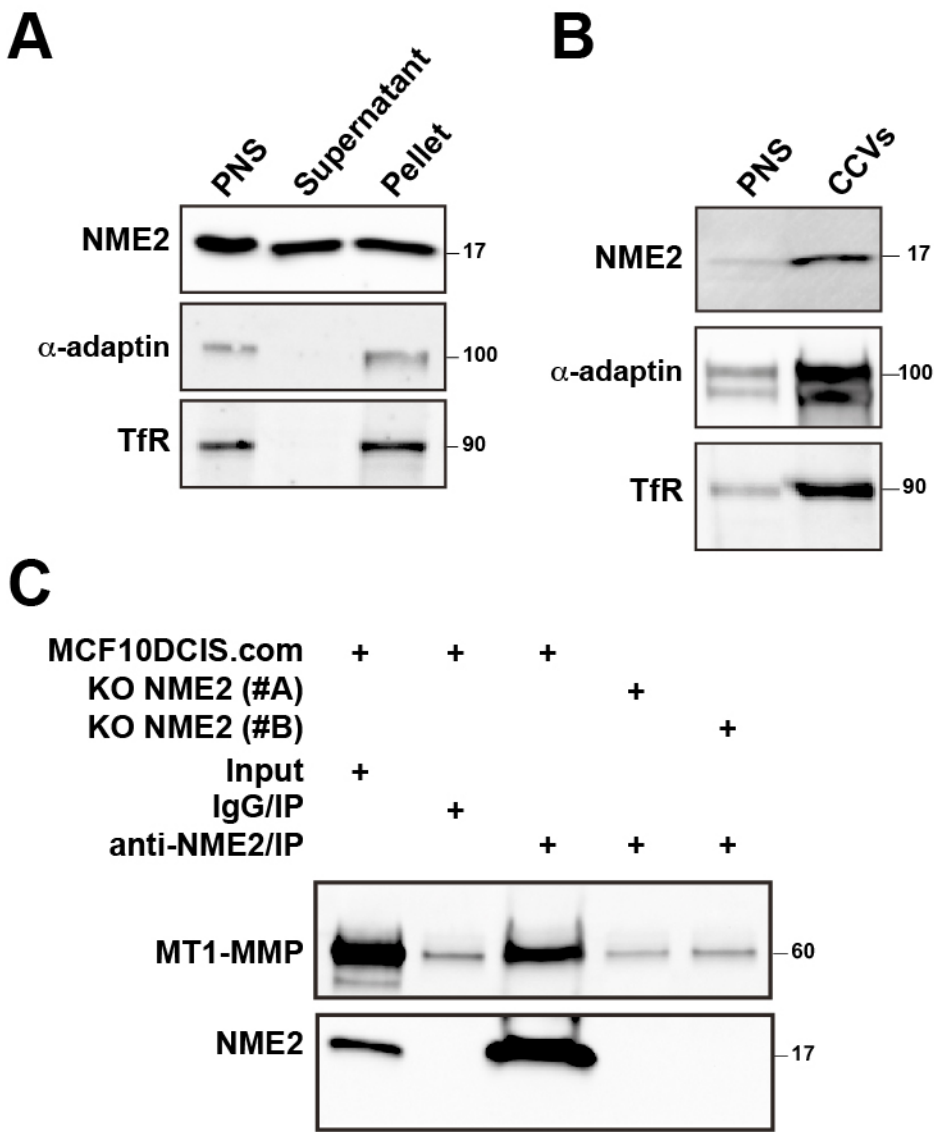

Supplement: Supplementary file 1 — Supplemental material [file 41388_2021_1826_MOESM1_ESM.pdf]
